# Cerebrospinal fluid-derived small extracellular vesicles reveal a compartment-specific diagnostic signature for NSCLC leptomeningeal metastasis

**DOI:** 10.1186/s12951-026-04847-8

**Published:** 2026-08-12

**Authors:** Peng-peng Kuang, Yang-si Li, Ting Hu, Mei-mei Zheng, Jia-tao Zhang, Kai Yin, Jin-ji Yang, Xue-ning Yang, Qing Zhou, Hai-yan Tu, Yi-long Wu, Wen-zhao Zhong, Ben-yuan Jiang

**Affiliations:** 1 Guangdong Lung Cancer Institute, Guangdong Cardiovascular Institute, Guangdong Provincial People’s Hospital, Guangdong Academy of Medical Sciences, Southern Medical University, Guangzhou, China; 2 Guangdong Provincial Key Laboratory of Translational Medicine in Lung Cancer, Guangdong Provincial People’s Hospital, Guangdong Academy of Medical Sciences, Southern Medical University, Guangzhou, China; 3 Department of Endoscopy, State Key Laboratory of Oncology in South China, Guangdong Provincial Clinical Research Center for Cancer, Sun Yat-Sen University Cancer Center, Guangzhou, China

**Keywords:** Small extracellular vesicles, MiRNA, NSCLC, Leptomeningeal metastasis, Biomarkers

## Abstract

**Background:**

Leptomeningeal metastasis (LM) is a devastating complication of non-small cell lung cancer (NSCLC), yet timely diagnosis remains challenging because conventional approaches, including magnetic resonance imaging (MRI) and cerebrospinal fluid (CSF) cytology, have limited sensitivity, particularly at initial presentation. Small extracellular vesicles (sEVs), as nanoscale carriers of protected regulatory cargo, have emerged as promising substrates for liquid biopsy. However, in anatomically compartmentalized central nervous system malignancies, the extent to which disease-associated sEV-miRNA signals are preserved across biofluids remains unclear.

**Methods:**

We profiled sEV-associated miRNAs by small RNA sequencing in CSF and plasma samples from NSCLC patients with clinically adjudicated LM status (NC, LM−, and LM+), including paired specimens where available. Biofluid-specific miRNA programs were characterized using weighted gene co-expression network analysis (WGCNA), and diagnostic models were developed using machine learning with Random Forest and LASSO-based feature selection. Model performance was evaluated in an independent validation cohort relative to final adjudicated LM status and compared with initial and cumulative MRI and CSF cytology assessments.

**Results:**

CSF-derived sEV-associated miRNAs showed markedly stronger disease-stratification capacity than plasma-derived signals (ARI = 0.813 vs. 0.255; cluster purity = 0.868 vs. 0.694), revealing profound biofluid asymmetry. Network analysis identified a dominant LM-associated module in CSF (*r* = 0.74, *P* = 9 × 10⁻⁷) with limited preservation in plasma, indicating that LM-related vesicle-associated molecular programs are compartment-restricted. From this CSF-specific nanoscale vesicle signature, we derived a four-miRNA diagnostic panel comprising hsa-let-7e-5p, hsa-miR-30d-5p, hsa-miR-486-5p, and hsa-miR-375. In the independent validation cohort, the single-sample sEV-miRNA panel achieved an AUC of 0.935, with 86.4% sensitivity and 87.5% specificity. By comparison, initial MRI and initial CSF cytology showed sensitivities of 50.0% and 77.3%, respectively, whereas cumulative MRI and cumulative CSF cytology showed sensitivities of 70.0% and 86.4%, respectively.

**Conclusions:**

CSF-derived sEV-associated miRNAs encode an LM-proximal molecular program that is not faithfully captured in plasma, underscoring the importance of biofluid compartmentalization in nanobiotechnology-based biomarker discovery. Our findings establish a compartment-aware sEV-miRNA diagnostic strategy for accurate LM detection and provide a conceptual framework for extracellular vesicle-based molecular diagnostics in central nervous system malignancies.

**Graphical Abstract:**

**Figure:**
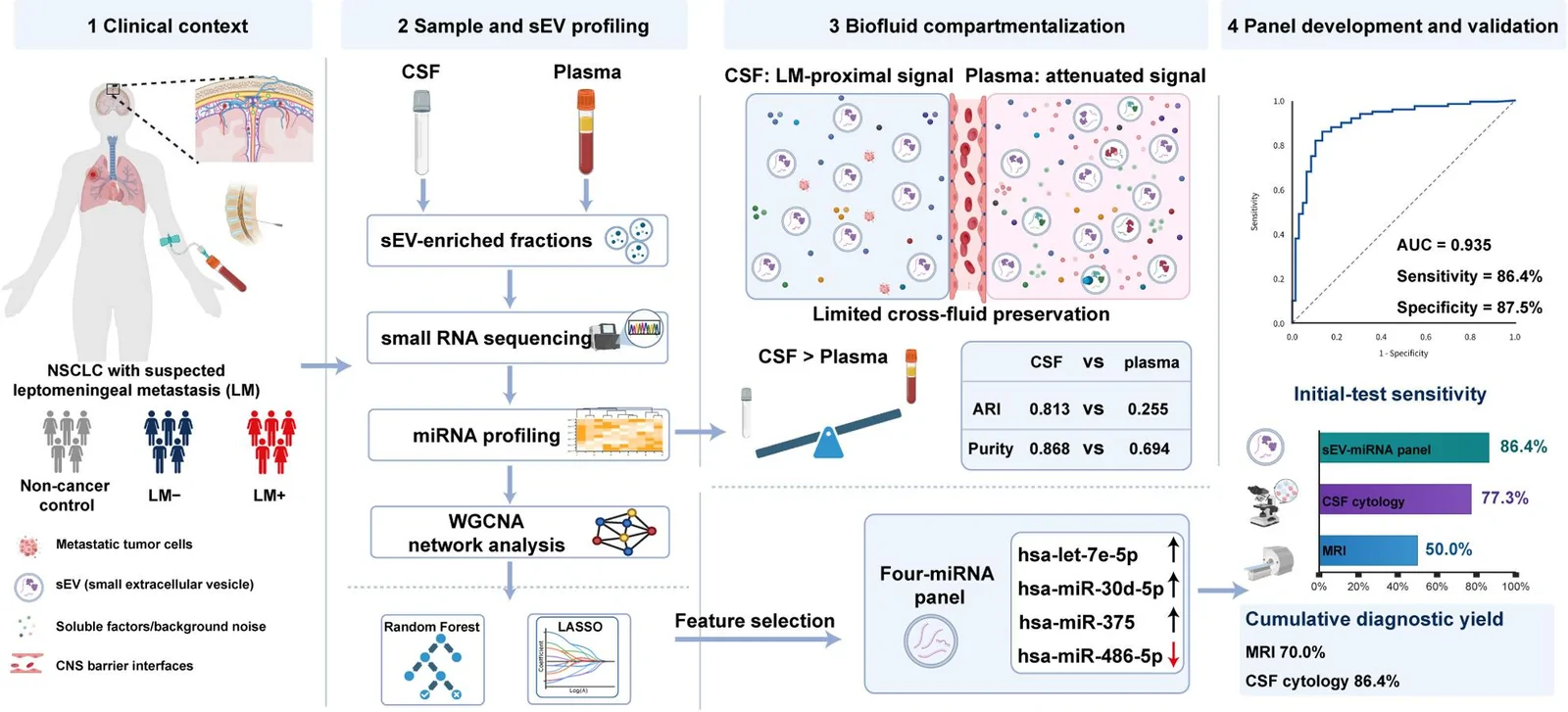

**Supplementary Information:**

The online version contains supplementary material available at https://doi.org/10.1186/s12951-026-04847-8.

## Introduction

Leptomeningeal metastasis (LM), defined as the dissemination of malignant cells into the subarachnoid space, represents a devastating complication of solid tumors, particularly in non-small cell lung cancer (NSCLC), breast cancer, and melanoma [1]. In NSCLC, the overall incidence of LM is approximately 3–5% [2], with a higher incidence reported in patients harboring epidermal growth factor receptor (EGFR) mutations (up to 9%) [3]. Despite therapeutic interventions, the clinical outlook remains poor, with median survival ranging between 3 and 11 months [3, 4]. A major bottleneck in LM management is the limited performance of current diagnostic standards. Clinical practice primarily relies on contrast-enhanced brain magnetic resonance imaging (MRI) and cerebrospinal fluid (CSF) cytology [5]. MRI often yields equivocal results in early-stage disease, whereas CSF cytology, the traditional “gold standard,” has low initial sensitivity and often requires repeated lumbar punctures to achieve a definitive diagnosis [6]. There is therefore an urgent need to identify accessible biomarkers and develop novel diagnostic strategies for LM.

Liquid biopsy strategies, including circulating tumor cells (CTCs), circulating tumor DNA (ctDNA), circulating RNAs, and small extracellular vesicles (sEVs), have expanded opportunities for minimally invasive cancer detection and monitoring [7, 8]. Among these analytes, sEVs are nanoscale membrane-bound particles enriched with nucleic acids and other bioactive cargo, acting as critical mediators of intercellular communication during cancer progression [9, 10]. sEV-associated microRNAs (miRNAs) mirror the molecular state of their parental cells and are protected from enzymatic degradation by the vesicular lipid bilayer, conferring high stability in biofluids [11]. These properties make sEV-associated miRNAs attractive candidates for nanobiotechnology-enabled molecular diagnostics. Blood-based sEV-miRNA signatures have already demonstrated clinical utility in the early detection of systemic malignancies, such as gastric cancer and colorectal cancer [12–14]. However, in central nervous system (CNS) malignancies and metastatic complications, disease biology is strongly shaped by anatomical and physiological compartmentalization. Accumulating evidence suggests that metastatic tumor cells release miRNA-containing sEVs capable of modulating endothelial integrity and disrupting the blood-brain barrier (BBB) [15, 16]. For LM, however, it remains unclear whether disease-associated sEV-miRNA signals are detectable in peripheral blood or are preferentially preserved in CSF [17]. Given the restrictive role of CNS barrier systems in limiting the systemic dissemination of brain- and meningeal-derived signals [18], plasma-derived sEV-associated miRNAs may not provide the same diagnostic fidelity as those obtained directly from CSF.

In this study, we systematically compared sEV-associated miRNA profiles in CSF and plasma from NSCLC patients with clinically adjudicated LM status. By integrating small RNA sequencing with weighted gene co-expression network analysis (WGCNA) and machine learning–based modeling, we aimed to define compartment-specific molecular features of LM and evaluate the relative diagnostic value of sEV-derived signals across biofluids. Through this approach, we aimed to establish a compartment-aware framework for extracellular vesicle-based biomarker development in CNS malignancies and to identify candidate CSF-derived miRNA signatures for LM detection.

## Materials and methods

### Patients and sample collection

A total of 79 participants were enrolled in this study and divided into a training cohort (*n* = 41) and an independent validation cohort (*n* = 38). The training cohort included 29 patients with stage IV NSCLC, four non-cancer patients, and eight healthy volunteers, while the validation cohort consisted of 38 patients with stage IV NSCLC. Healthy volunteers were defined as individuals without known malignancy or neurological disease and provided plasma samples only. CSF was not collected from healthy volunteers because lumbar puncture solely for research purposes was not ethically justified. Non-cancer patients were defined as individuals who underwent CSF collection for clinical evaluation of non-malignant neurological conditions and had no evidence of malignancy or leptomeningeal metastasis after clinical assessment. These non-cancer patients served as the available non-malignant CSF control group. None of the non-cancer patients had acute traumatic brain injury, intracranial hemorrhage, or other acute CNS injury at the time of CSF collection. NSCLC patients with suspected intracranial metastasis underwent a standardized diagnostic workflow, including contrast-enhanced brain MRI and CSF cytological examination. MRI scans performed within 30 days of CSF collection were reviewed by at least one radiologist with expertise in neuro-oncology imaging. For NSCLC patients, LM status was determined by final clinical adjudication based on all available clinical information, including contrast-enhanced MRI, CSF cytology, meningeal biopsy when available, and longitudinal clinical follow-up. The sEV-miRNA panel was not used for LM adjudication. Patients were classified as LM positive (LM+) if they had malignant cells detected in CSF, biopsy-confirmed meningeal involvement, or clinically adjudicated LM based on typical radiographic findings and clinical course. Patients without evidence of LM after MRI, CSF cytology, and longitudinal follow-up were classified as LM negative (LM−). Intraparenchymal brain metastasis (IM) was evaluated by MRI.

**Fig. 1:**
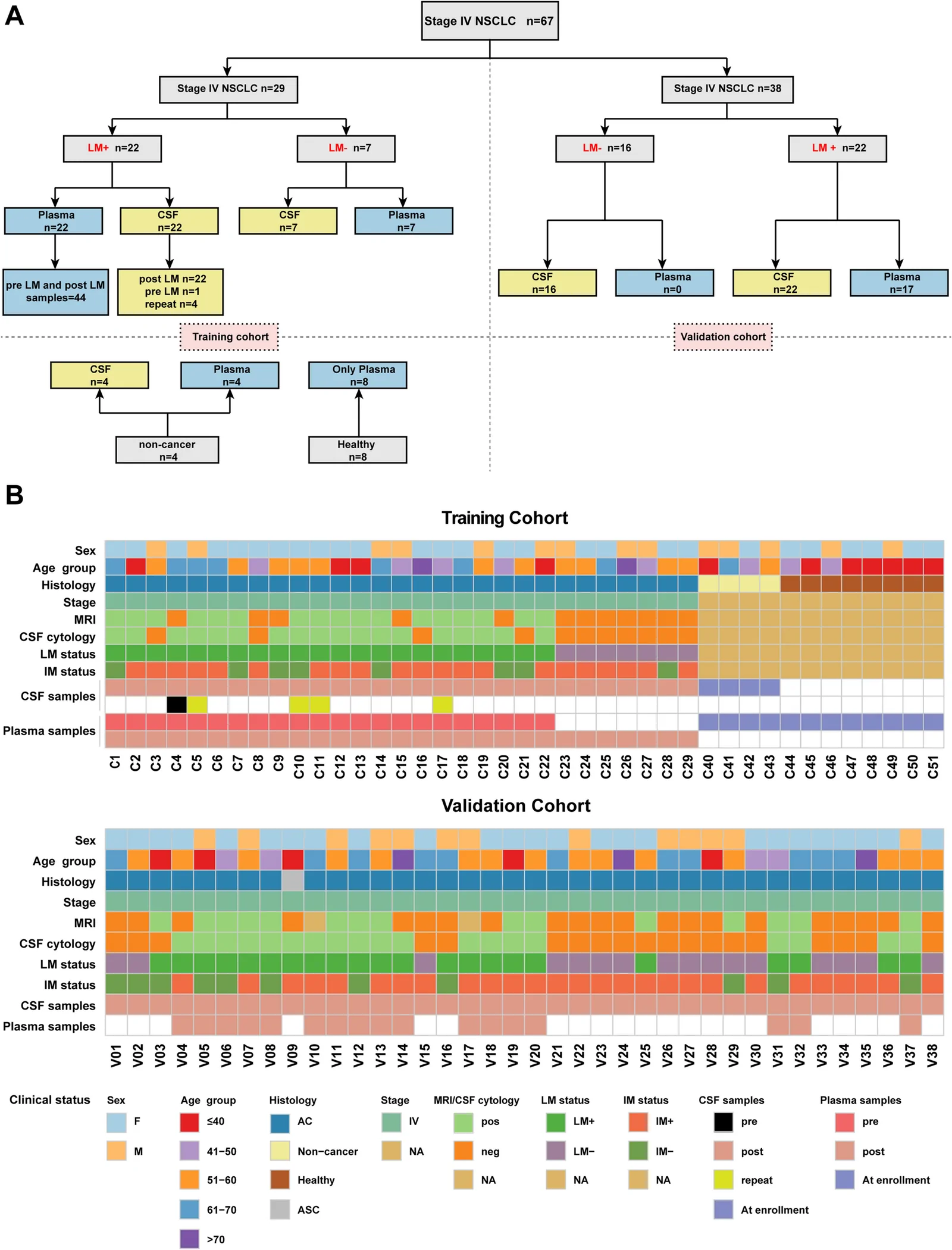
Study cohorts, biofluid sampling workflow, and clinical feature overview. **(A)** Study design and biofluid sampling scheme. The schematic summarizes CSF and plasma collection in LM + and LM− groups, including paired plasma samples collected before and after LM diagnosis and repeat CSF sampling in a subset of LM+ cases. Control groups included non-cancer controls (CSF and plasma) and healthy controls (plasma only). **(B)** Clinical-feature matrix for the training (top) and validation (bottom) cohorts. Columns represent individual participants and rows indicate clinical variables, including sex, age group, histology, stage, cumulative MRI status, cumulative CSF cytology, LM status, and IM status. Sample availability and time points are shown in the “CSF samples” and “Plasma samples” rows. Abbreviations: LM, leptomeningeal metastasis; CSF, cerebrospinal fluid; IM, intraparenchymal brain metastasis

A total of 156 biospecimens (plasma and CSF) were collected, and the overall sampling scheme and timeline are illustrated in Fig. 1. Plasma samples were obtained during routine clinical care. In the training cohort, 22 NSCLC patients were classified as LM + and seven as LM−. For LM+ patients, paired plasma samples were collected before and after LM diagnosis, and CSF samples were obtained after LM confirmation. One LM+ patient additionally contributed a pre-LM CSF sample collected 321 days prior to clinical diagnosis, and four LM+ patients underwent repeat lumbar punctures, yielding a total of 27 CSF samples associated with LM+ disease. For LM− patients, CSF and plasma samples were collected according to the same clinical workflow. In the control groups, paired CSF and plasma samples were collected from four non-cancer patients, whereas plasma samples alone were obtained from eight healthy volunteers. Consequently, the training cohort yielded 38 CSF samples and 63 plasma samples. In the validation cohort, CSF samples were collected from all 38 stage IV NSCLC patients, and paired plasma samples were available for 17 patients. Baseline demographic and clinicopathological characteristics are summarized in Supplementary Tables S1 and S2. All participants provided written informed consent, and the study protocol was approved by the Ethics Committee of Guangdong Provincial People’s Hospital.

### Isolation of sEV-enriched fractions from CSF and plasma

CSF samples were centrifuged at 2,000 × g for 20 min at 4 °C, followed by centrifugation at 10,000 × g for 30 min at 4 °C. The supernatant was then ultracentrifuged at 100,000 × g for 90 min at 4 °C. The resulting pellet was washed with ice-cold PBS and ultracentrifuged again at 100,000 × g for an additional 90 min. The final pellet was resuspended in PBS and used as the CSF-derived sEV-enriched fraction for downstream analyses. Plasma-derived sEV-enriched fractions were isolated using size-exclusion chromatography (SEC) with qEV columns (Izon Science). Briefly, plasma samples were centrifuged at 2,000 × g for 10 min and 10,000 × g for 20 min at 4 °C to remove debris and large particles. The supernatant was loaded onto pre-equilibrated qEV columns and eluted with PBS. sEV-enriched fractions were collected according to the manufacturer’s instructions, pooled, and concentrated using 100 kDa centrifugal ultrafiltration tubes (Merck Millipore).

Different isolation strategies were applied to CSF and plasma to accommodate biofluid-specific properties, including differences in protein content, lipoprotein burden, and vesicle abundance. To avoid overinterpretation of purity, all preparations are therefore referred to as sEV-enriched fractions.

### Nanoparticle tracking analysis (NTA)

Nanoparticle tracking analysis (NTA) was performed to characterize the particle concentration and size distribution of sEV-enriched fractions. Samples were diluted in PBS to achieve particle concentrations within the optimal measurement range (5–10 × 10⁸ particles/mL). Measurements were conducted using a NanoSight NS300 system (Malvern, UK) with standardized acquisition settings (camera level 15, detection threshold 3). For each sample, three independent recordings were acquired and analyzed using the manufacturer’s software, and results were averaged to determine particle size distribution (nm) and concentration (particles/mL).

### Transmission electron microscopy (TEM)

Transmission electron microscopy (TEM) was performed to assess the morphology of sEV-enriched fractions. Samples were fixed in 2% glutaraldehyde in PBS and applied onto carbon-coated copper grids. After washing to remove excess salts, grids were negatively stained with 3% uranyl acetate. Images were acquired using a Hitachi H7650 transmission electron microscope (Tokyo, Japan) operating at an accelerating voltage of 100 kV.

### Western blot

Western blotting was performed to assess the enrichment of canonical extracellular vesicle–associated protein markers in sEV-enriched fractions. The protein concentration of sEV samples was determined using the Pierce^™^ BCA Protein Assay Kit (Thermo Fisher Scientific, Rockford, USA). Proteins were mixed with SDS loading buffer and denatured at 95 °C for 10 min. Samples were separated by sodium dodecyl sulfate polyacrylamide gel electrophoresis (SDS-PAGE) and transferred to a polyvinylidene difluoride (PVDF) membrane (Roche Diagnostics, Mannheim, Germany). Membranes were blocked with 5% skim milk in TBST at room temperature for 1 h and incubated overnight at 4 °C with primary antibodies against CD9 (rabbit monoclonal, CST, 13403 S, 1:1,000), CD63 (Proteintech, 25682-1-AP, 1:400), TSG101 (Proteintech, 28283-1-AP, 1:2,000), and calnexin (Proteintech, 10427-2-AP, 1:1,000). After three washes with TBST, membranes were incubated with an HRP-conjugated anti-rabbit IgG secondary antibody at room temperature for 1 h. Bands were visualized using the ECL Plus detection reagent (Merck Millipore, Billerica, USA) and imaged with the ChemiDoc Imaging System (Bio-Rad Laboratories, Hercules, CA, USA).

### RNA isolation and small RNA sequencing library preparation

Total RNA was extracted from CSF- and plasma-derived sEV-enriched fractions using the miRNeasy Serum/Plasma Kit (QIAGEN) according to the manufacturer’s instructions. RNA concentration and size distribution were assessed using a Qubit 3.0 Fluorometer (Thermo Fisher Scientific) and an Agilent 2100 Bioanalyzer (Agilent Technologies, Santa Clara, CA, USA), respectively. Small RNA sequencing libraries were prepared from the isolated RNA using NEBNext^®^ Small RNA Library Prep Set for Illumina^®^ (NEB Biolabs) following the manufacturer’s protocol. Briefly, 3′ and 5′ adapters were ligated to the RNA, followed by 17 cycles of PCR amplification. The PCR products were separated by gel electrophoresis on a 6% Novex^®^ TBE PAGE gel (Thermo Fisher Scientific), and DNA fragments corresponding to the small RNA fraction (approximately 140–150 bp) were excised and recovered. The purified libraries were quantified using the Qubit 3.0 and validated on the Agilent 2100 Bioanalyzer. Libraries from different CSF and plasma samples were pooled at equimolar concentrations and sequenced on an Illumina HiSeq 4000 platform by Nanjing Geneseeq Technology Inc. (Nanjing, China).

### miRNA-Seq data processing and differential expression analysis

Raw miRNA sequencing reads from sEV-enriched CSF and plasma samples were processed using miRDeep2 for 3′ adapter trimming and length filtering (18–26 nt), followed by quantification against miRBase (v21). Low-abundance miRNAs were filtered to reduce noise. Library sizes were normalized using the TMM method. Differential expression (DE) was assessed using negative-binomial generalized linear models with quasi-likelihood testing in edgeR, with significance defined as FDR < 0.05 and |log2FC| > 1. For CSF samples, comparisons were conducted between LM + and a combined control group (LM − and non-cancer), supported by minimal transcriptional differences between these control subsets. For plasma samples, we performed four cross-sectional contrasts (Pre-LM + vs. LM−/LTF; Post-LM + vs. LM−/LTF) and paired longitudinal analyses (Post-LM vs. Pre-LM; Post-LM plasma vs. matched CSF), the latter using patient identity as a blocking factor. Highly variable miRNAs were used for principal component analysis and hierarchical clustering (Euclidean distance, Ward.D2 linkage). Clustering performance was evaluated using the adjusted Rand index and cluster purity. For differentially expressed miRNAs, target prediction was performed with TargetScan Human, retaining predicted targets within the strongest 5% of context ++ scores for each miRNA. Gene Ontology (GO) and Kyoto Encyclopedia of Genes and Genomes (KEGG) pathway enrichment analyses were conducted using the TargetScan background gene set, and pathways were ranked by FDR-adjusted P values.

### Co-expression network construction and cross-biofluid comparison

Weighted gene co-expression network analysis (WGCNA) was performed separately for CSF and plasma datasets to identify coordinated miRNA programs associated with LM. WGCNA module construction and hub-miRNA identification were restricted to representative samples from the training cohort. Specifically, the pre-LM CSF sample from patient C4 and repeat post-LM CSF samples from patients C5, C10, C11, and C17 were excluded from WGCNA module construction to avoid repeated sampling from the same patient during network analysis. For CSF and plasma datasets, low-abundance miRNAs were filtered, and expression values were normalized using the TMM method and transformed to log2 counts per million. Networks were constructed using an unsigned approach, with soft-thresholding power selected based on scale-free topology criteria. Modules were detected by dynamic tree cutting and merged based on eigengene similarity. Module eigengenes were correlated with ordinal LM status using Pearson correlation. Candidate hub miRNAs were defined based on absolute module membership (MM > 0.8) and absolute gene significance (GS > 0.5). Differences in module eigengene expression across NC, LM−, and LM+ groups were evaluated using one-way ANOVA followed by Tukey’s post hoc test (two-sided). Cross-biofluid correspondence was assessed in patients with paired CSF and plasma samples using: (i) Pearson correlations between all CSF and plasma module eigengenes; (ii) permutation-based module preservation analysis with Zsummary statistics; and (iii) GSVA-derived module activity scores correlated across paired CSF and plasma samples.

### Machine learning–based biomarker modeling

Candidate features for LM classification were derived from hub miRNAs identified from the training-cohort CSF WGCNA analysis described above. The training cohort was used for feature selection, model development, internal performance evaluation, and cutoff determination, whereas the independent validation cohort was reserved for external evaluation of the finalized model. Several supervised classification algorithms were evaluated using these candidate miRNAs, including Random Forest, logistic regression, decision tree, k-nearest neighbors, and support vector machine classifiers. Model performance was assessed using a bootstrap-based internal resampling framework implemented in the mlr3 R package. Based on this internal evaluation, Random Forest showed strong discriminative performance and was used for permutation-based variable-importance ranking. Feature selection was further refined by LASSO logistic regression. Candidate miRNAs prioritized by Random Forest importance ranking and retained by LASSO logistic regression were used to define a parsimonious four-miRNA panel. A logistic regression model incorporating these four miRNAs was then fitted in the training cohort to generate a panel probability score for each sample. The threshold for classifying samples as panel-positive or panel-negative was determined in the training cohort by maximizing the Youden index on the receiver operating characteristic (ROC) curve. The finalized four-miRNA panel, logistic regression coefficients, and training-derived cutoff were then applied unchanged to the independent validation cohort. The overall workflow is summarized in Fig. 6A.

### Statistical analysis

All analyses were performed in R (version 4.4.2). Unless otherwise stated, statistical significance for group-wise comparisons was assessed using two-sided tests. For high-dimensional analyses, multiple testing was controlled using the Benjamini–Hochberg procedure. For differential expression analyses, statistical significance was defined as FDR < 0.05 and |log2FC| > 1. In paired longitudinal plasma analyses, a relaxed exploratory threshold (nominal *P* < 0.05 and |log2FC| > 0.58) was additionally applied. Sensitivity, specificity, and corresponding 95% confidence intervals were calculated relative to the final adjudicated LM status. The 95% confidence intervals for sensitivity and specificity were estimated using the Wilson score method. The specific statistical methods used for each analysis are described in the corresponding Methods sections.

## Results

### Patient demographics and clinicopathological characteristics

The overall study design, cohort structure, and biospecimen distribution are shown in Fig. 1. Briefly, 79 participants were included, comprising a training cohort (*n* = 41) and an independent validation cohort (*n* = 38). The training cohort included 29 patients with stage IV NSCLC, four non-cancer patients, and eight healthy volunteers, whereas the validation cohort included 38 independent stage IV NSCLC patients. In the overall training cohort, 65.9% were female, and the mean age was 50.9 ± 13.4 years. Among the 29 NSCLC patients in the training cohort, all had adenocarcinoma. Among the 22 LM+ patients in the training cohort, cumulative MRI and CSF cytology identified 17 (77.3%) and 18 (81.8%) positive cases, respectively, with both modalities positive in 14/22 (63.6%). One patient had negative cumulative MRI and CSF cytology findings but was ultimately confirmed to have LM by meningeal biopsy, highlighting the need for multimodal and longitudinal diagnostic assessment. Intraparenchymal brain metastases were observed in 72.4% (21/29) of the NSCLC training cases. The validation cohort comprised 22 LM + and 16 LM− stage IV NSCLC patients. The sex distribution was broadly similar to that of the training cohort, and the mean age was 56.2 ± 10.7 years. Adenocarcinoma remained the predominant histological subtype (37/38, 97.4%). Detailed cohort characteristics and biospecimen distribution are summarized in Fig. 1 and Supplementary Tables S1 and S2.

### Isolation and characterization of CSF- and plasma-derived sEV-enriched fractions

**Fig. 2:**
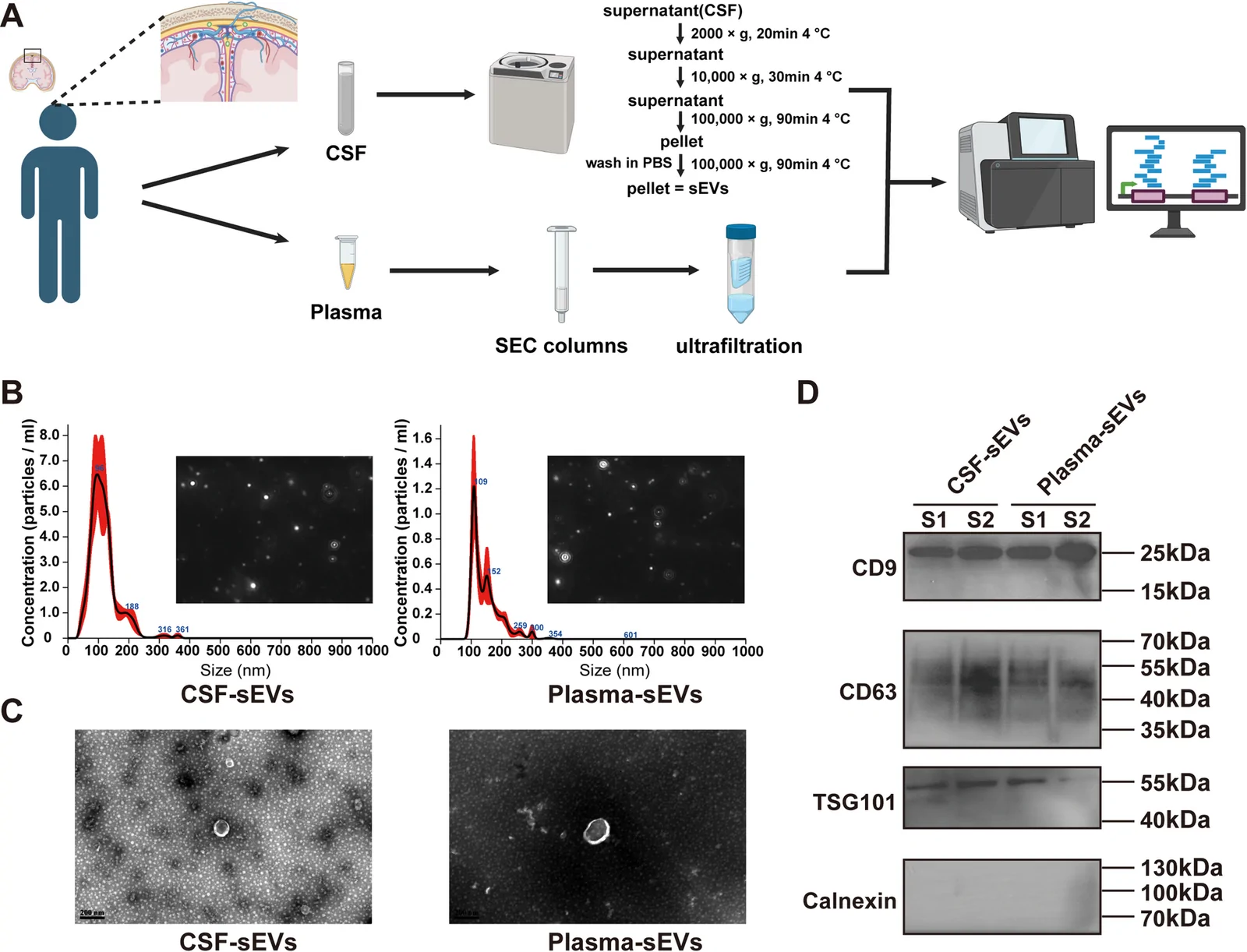
Isolation and characterization of sEV-enriched fractions from CSF and plasma. **(A)** Schematic overview of the experimental workflow for isolation of sEV-enriched fractions from CSF and plasma. **(B)** Nanoparticle tracking analysis (NTA) showing representative particle size distributions from CSF (left) and plasma (right). Insets show representative particle-tracking visualization fields. **(C)** Transmission electron microscopy (TEM) images of CSF- and plasma-derived preparations, showing membrane-bound vesicular structures consistent with extracellular vesicles. Scale bar, 200 nm. **(D)** Western blot analysis of EV-associated protein markers (CD9, CD63, and TSG101) in CSF- and plasma-derived samples from representative cases (S1 and S2). The absence of calnexin suggests minimal contamination from intracellular components. Figure 2A was created with BioRender.com

sEV-enriched fractions were isolated from CSF samples using ultracentrifugation and from plasma samples using SEC (Fig. 2A). To assess the quality of the isolates, we performed multiparametric characterization following the MISEV2018 guidelines [19]. Nanoparticle tracking analysis demonstrated that both CSF- and plasma-derived sEV-enriched preparations displayed a predominant size peak at approximately 100 nm, with minor larger subpopulations extending to approximately 150–200 nm (Fig. 2B). Morphological integrity was assessed via TEM, which displayed characteristic cup-shaped or spherical membrane-bound structures in both preparations (Fig. 2C). To further confirm the biochemical identity of the vesicles, immunoblotting was employed to assess the enrichment of protein markers. Both CSF- and plasma-derived sEV-enriched fractions showed expression of canonical sEV-associated markers, including the tetraspanins (CD9 and CD63) and the cytosolic protein TSG101 (Fig. 2D). Crucially, the endoplasmic reticulum marker calnexin was not detected in these fractions, indicating minimal intracellular organelle contamination (Fig. 2D). Collectively, these physical, morphological, and biochemical analyses support successful isolation of sEV-enriched fractions suitable for downstream small RNA sequencing and biomarker discovery.

### Characterization of the sEV-associated miRNA Landscape in CSF

**Fig. 3:**
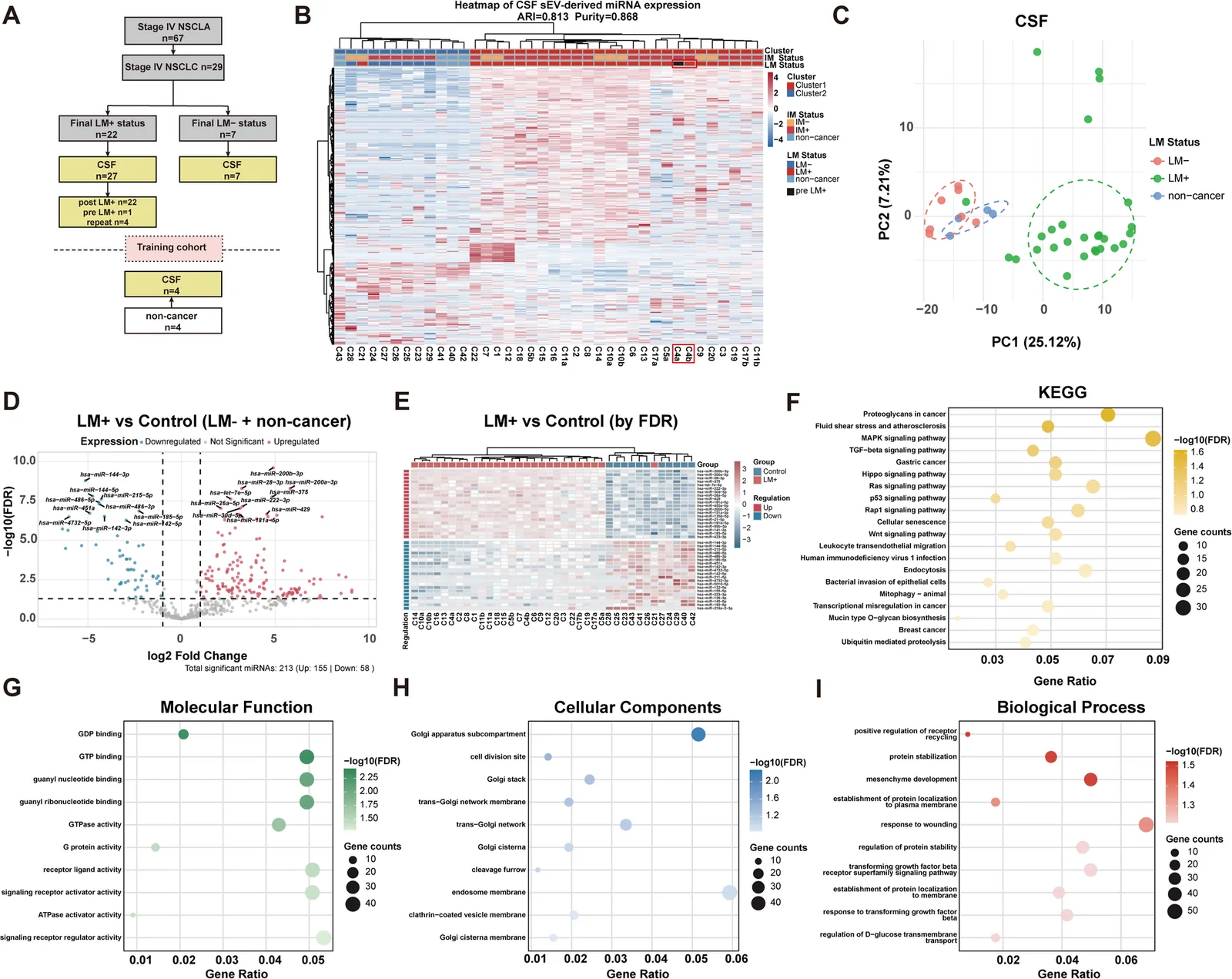
CSF sEV-associated miRNA profiling reveals an LM-associated signature and functional programs. **(A)** Overview of CSF samples included in the training cohort. **(B)** Unsupervised hierarchical clustering heatmap of CSF sEV-associated miRNA expression. Clustering performance relative to LM status is shown (ARI = 0.813; purity = 0.868). **(C)** Principal component analysis (PCA) of CSF sEV-associated miRNA expression, colored by LM status. **(D)** Volcano plot of differential sEV-associated miRNA expression in LM+ versus control (LM − and non-cancer). Significant miRNAs are defined as FDR < 0.05 and |log2FC| > 1. Labeled miRNAs denote the most statistically significant changes. **(E)** Heatmap showing the top differentially expressed sEV-associated miRNAs (ranked by FDR) for LM + vs. Control. **(F)** KEGG pathway enrichment of high-confidence TargetScan-predicted targets associated with downregulated differentially expressed sEV-associated miRNAs. Bubble plot shows the top enriched pathways; dot size represents gene counts, and color indicates significance (− log10 FDR). **(G–I)** Gene Ontology (GO) enrichment of predicted targets of downregulated differentially expressed sEV-associated miRNAs, shown as bubble plots for Molecular Function **(G)**, Cellular Component **(H)**, and Biological Process **(I)**. Dot size indicates gene counts and color indicates significance (− log10 FDR)

To delineate the sEV-associated miRNA signatures linked to leptomeningeal metastasis, we performed small RNA sequencing on 38 CSF samples from the training cohort (Fig. 3A). Unsupervised hierarchical clustering of the miRNA expression profiles effectively segregated the cohort into two primary clusters: a metastatic group (LM+) and a control group (comprising LM − and non-cancer specimens) (Fig. 3B). The clustering performance was robust, as quantified by an Adjusted Rand Index (ARI) of 0.813 and cluster purity of 0.868, with only one LM+ sample misclassified. Notably, the longitudinal pre-diagnostic sample from patient C4 (C4a), obtained 321 days prior to LM diagnosis, clustered closely with its matched post-diagnosis sample (C4b). Although based on a single case, this finding suggests that broad CSF sEV-associated miRNA alterations may be detectable before clinical LM confirmation in selected patients. In contrast to LM status, CSF sEV-associated miRNA profiles did not clearly differentiate patients with or without intraparenchymal brain metastases (IM), suggesting that the dominant CSF sEV-miRNA signal was more closely associated with leptomeningeal involvement than with the presence of parenchymal brain metastasis alone (Fig. 3B). Consistent with the clustering results, PCA showed clear separation of LM+ samples from the LM − and non-cancer groups along the major principal components (PC1 = 25.12%, PC2 = 7.21%). Conversely, LM − and non-cancer samples largely overlapped in the PCA space, suggesting similar global sEV-associated miRNA expression patterns between these non-LM groups (Fig. 3C). This overlap, together with minimal differential expression between LM − and non-cancer controls (Supplementary Fig. S1A), supported consolidation of these groups into a single Control group for downstream differential expression analysis.

Differential expression analysis identified 213 significant miRNAs (FDR < 0.05, |log2FC| > 1), consisting of 155 upregulated and 58 downregulated miRNAs in LM+ compared with Control (Fig. 3D–E). Heatmap analysis of the top-ranked DE miRNAs exhibited a coherent bidirectional expression pattern, with LM + and control samples displaying distinct group-specific profiles (Fig. 3E). Among the most significantly upregulated features were members of the miR-200 family (miR-200a/b-3p, miR-200b-5p, and miR-141-3p), known mediators of epithelial-mesenchymal transition (EMT) and metastatic colonization [20, 21]. Conversely, the tumor-suppressive miR-144/451 cluster showed marked downregulation in the LM+ group. Internal subgroup comparisons confirmed that LM+ samples exhibited substantial miRNA dysregulation relative to both LM − and non-cancer controls, whereas LM − and non-cancer samples showed minimal differences (Supplementary Fig. S1A–C), indicating that the dominant miRNA expression shift is specifically associated with leptomeningeal dissemination.

Functional enrichment analysis of high-confidence TargetScan-predicted targets provided insights into biological programs potentially associated with LM. For targets associated with downregulated miRNAs, KEGG pathway analysis highlighted a predominant enrichment in classical signaling cascades including MAPK, TGF-beta, Ras, and Wnt signaling (Fig. 3F). Gene Ontology (GO) enrichment characterized the functional landscape: Molecular Function was enriched for GTPase activity and nucleotide binding, Cellular Component for Golgi-related compartments and endosomal membranes, and Biological Process for protein localization, mesenchyme development, and regulation of protein stability (Fig. 3G–I). In parallel, targets associated with upregulated miRNAs were notably enriched in metabolic processes—such as xenobiotic metabolism, pentose and glucuronate interconversions, and steroid hormone biosynthesis (Supplementary Fig. S1D–G). This divergence suggests that LM-associated miRNA alterations may coordinate a dual mechanism: potentiating oncogenic signaling via downregulation of suppressive miRNAs, while simultaneously dampening specific metabolic and developmental differentiation programs via upregulated miRNAs.

Together, these results demonstrate that CSF sEV-associated miRNAs capture a coherent LM-associated molecular program.

### Characterization of the sEV-associated miRNA landscape in plasma

**Fig. 4:**
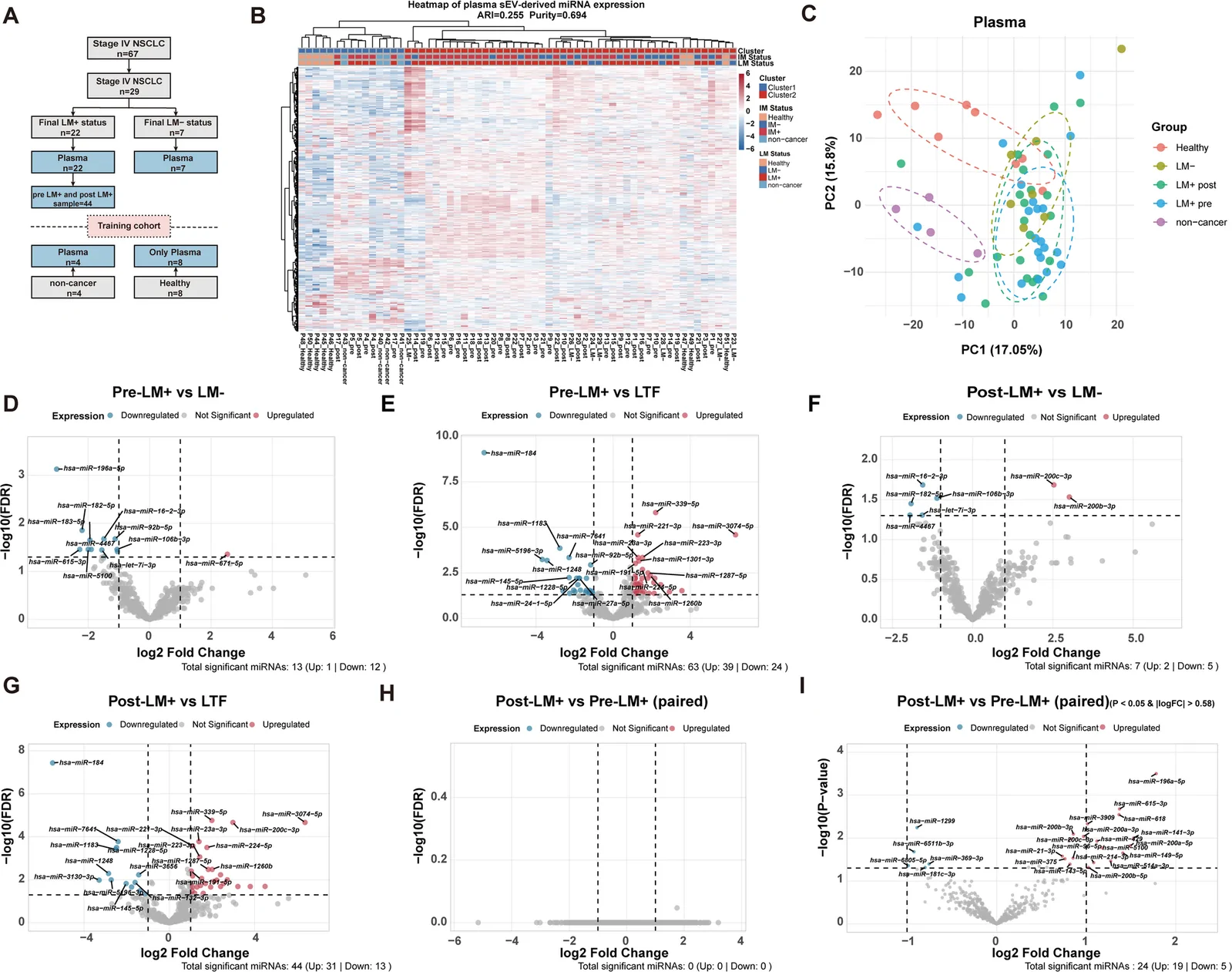
Plasma sEV-associated miRNA profiling reveals limited LM-associated signal. **(A)** Overview of plasma samples included in the training cohort. One sample was excluded during quality control, resulting in a final analytical cohort of 62 plasma samples. **(B)** Unsupervised hierarchical clustering heatmap of plasma sEV-associated miRNA expression. Clustering performance relative to LM status is shown (ARI = 0.255; purity = 0.694). **(C)** PCA of plasma sEV-associated miRNA profiles across pre-LM+, post-LM+, LM−, non-cancer, and healthy samples. **(D–G)** Volcano plots showing differential expression of sEV-associated miRNAs: **(D)** Pre-LM + vs. LM−, **(E)** Pre-LM + vs. LTF, **(F)** Post-LM + vs. LM−, and **(G)** Post-LM + vs. LTF. Labeled miRNAs denote the most statistically significant changes. **(H–I)** Differential analysis of paired longitudinal plasma samples (Post-LM vs. Pre-LM). **(H)** Using stringent thresholds (FDR < 0.05 and |log2FC| > 1), no differentially expressed miRNAs were detected. **(I)** Using relaxed exploratory criteria (*P* < 0.05 and |log2FC| > 0.58), a predominantly upregulated miRNA program was observed following LM onset. Abbreviations: LTF, leptomeningeal tumor-free

Given the superior accessibility of blood-based liquid biopsies, we characterized sEV-associated miRNA expression profiles in 62 quality-controlled plasma samples (out of 63 originally collected) to evaluate their diagnostic utility for LM (Fig. 4A). In contrast to the clear stratification observed in CSF, unsupervised hierarchical clustering of plasma samples showed only modest agreement with LM status, with an ARI of 0.255 and purity of 0.694 (Fig. 4B). PCA similarly revealed substantial overlap across clinical groups, though a subtle group-level displacement of LM-associated samples from the Control manifold was discernible (Fig. 4C). These results indicate that LM-related plasma sEV-associated miRNA signals are present but are weaker and more variable than those in CSF. Consistent with the unsupervised findings, differential expression analyses revealed limited discriminative power within the cancer-specific cohort. Compared with LM−, Pre-LM+ samples showed only 13 significant miRNAs, while the Post-LM + vs. LM− comparison identified merely 7 significant miRNAs, with changes largely skewed toward downregulation (Fig. 4D, F). In contrast, more prominent differences emerged when LM cases were compared with leptomeningeal tumor-free (LTF) controls, defined by pooling non-cancer and healthy plasma samples to provide a tumor-free baseline reference (Pre-LM + vs. LTF: 63 significant DE miRNAs; Post-LM + vs. LTF: 44 significant DE miRNAs) (Fig. 4E, G). This disparity implies that the apparent plasma signals largely reflect general malignancy-associated changes rather than LM-specific molecular events. Furthermore, in the paired longitudinal analysis (Post-LM vs. Pre-LM), no miRNAs reached statistical significance under the predefined stringent thresholds (FDR < 0.05 and |log2 FC| > 1) (Fig. 4H). Only upon applying relaxed exploratory criteria (*P* < 0.05 and |log2FC| > 0.58) were 24 miRNAs identified, predominantly upregulated (19 upregulated and 5 downregulated) (Fig. 4I), indicating that intra-patient dynamics of plasma sEV-associated miRNAs remain subtle throughout LM progression.

Collectively, these results indicate that plasma sEV-associated miRNAs provide weaker and less LM-specific signals for LM detection, supporting the prioritization of CSF for biomarker development.

### Comparative analysis of sEV-associated miRNA profiles between matched CSF and plasma samples

To further define differences between biofluid compartments, we performed paired analyses using matched CSF and plasma samples from the same patients. PCA showed a clear separation by biofluid origin, with CSF and plasma forming two distinct clusters (PC1 = 26.5% variance; Supplementary Fig. S2A). Differential expression analysis identified 308 significantly deregulated miRNAs (FDR < 0.05), indicating substantial divergence between compartments. CSF-derived sEV-associated miRNA profiles were enriched for epithelial-associated oncogenic signatures (e.g., miR-200 family), whereas plasma profiles were dominated by signals consistent with systemic and hematopoietic origins, including miR-223-3p and miR-126-3p (Supplementary Fig. S2B–C). Functional enrichment analysis further supported this compartmental distinction. Plasma-associated signals were linked to broad systemic pathways, including cancer signaling and endocytic processes (Supplementary Fig. S2D–G), whereas CSF-associated signals were enriched in metabolic and secretory pathways (Supplementary Fig. S2H–K). Together, these results demonstrate a pronounced compartment-specific divergence between CSF and plasma sEV-associated miRNA profiles, supporting the concept that CSF may better capture CNS-compartment-associated disease signals.

### CSF sEV-associated miRNA co-expression networks reveal a dominant LM-coupled module with limited cross-fluid preservation

**Fig. 5:**
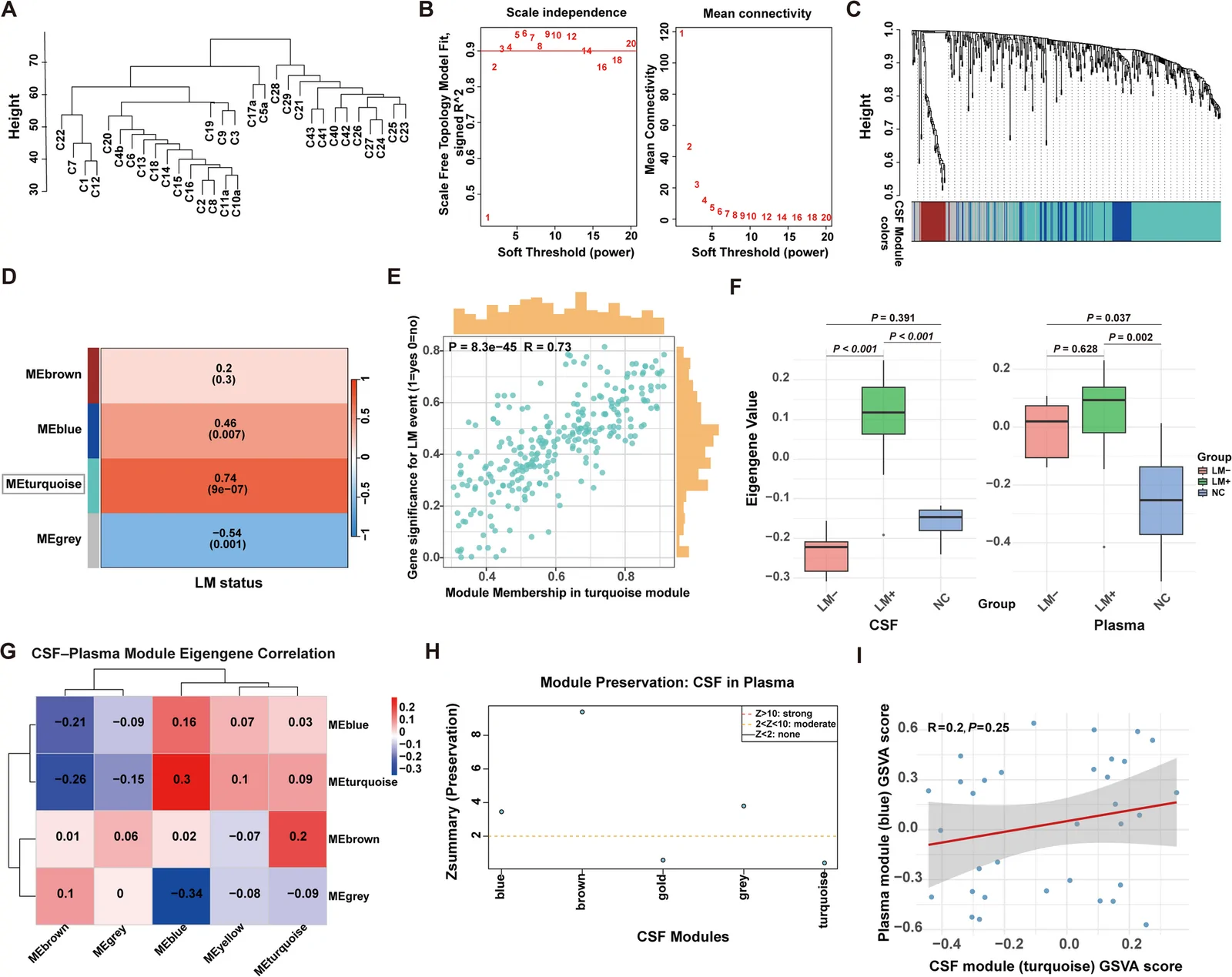
Weighted gene co-expression network analysis (WGCNA) of CSF sEV-associated miRNAs reveals an LM-associated module with limited cross-fluid preservation. **(A)** Sample clustering dendrogram of CSF samples. **(B)** Soft-thresholding analysis identifies power = 5 as the selected parameter for network construction. **(C)** Clustering dendrogram and module assignment illustrating discrete co-expression modules in CSF sEV-associated miRNAs. In WGCNA, each color-labeled module represents a cluster of miRNAs with similar co-expression patterns, and the module eigengene summarizes the overall expression behavior of that module. **(D)** Module–trait correlation heatmap showing associations between CSF module eigengenes and LM status; the turquoise module is highlighted as the dominant LM-associated CSF module. **(E)** Scatter plot of module membership (MM) versus gene significance (GS) for miRNAs in the CSF turquoise module. **(F)** Distribution of module eigengene expression across clinical strata (NC, LM−, LM+). The CSF turquoise module (left) shows a stepwise increase peaking in LM+ samples, whereas the plasma blue module (right) shows a weaker association across the same strata. **(G)** Cross-fluid module eigengene correlation matrix between CSF and plasma modules in paired patients. **(H)** Module preservation analysis using CSF as the reference dataset to assess preservation in plasma. Zsummary thresholds indicate strong preservation (Z > 10), moderate preservation (2 < Z < 10), or no/weak preservation (Z < 2). **(I)** Correlation of CSF turquoise and plasma blue module activities in paired samples using GSVA-based scores. Note: Pre-diagnostic CSF samples and repeat post-diagnosis CSF samples were excluded from WGCNA to avoid potential bias introduced by longitudinal sampling

To define coordinated sEV-associated miRNA programs linked to leptomeningeal metastasis, we performed WGCNA separately in matched CSF and plasma sEV-associated miRNA profiles from 33 patients spanning non-cancer (NC), LM−, and LM+ states. Initial sample clustering demonstrated stable expression patterns without outliers (Fig. 5A), and network construction yielded well-defined co-expression modules (Fig. 5B–C). In the CSF cohort, module-phenotype relationships highlighted a dominant LM-associated program, with the turquoise module showing the strongest positive association with LM status (Pearson *r* = 0.74, *P* = 9 × 10⁻⁷) (Fig. 5D). At the miRNA level, the turquoise module demonstrated a strong concordance between module membership and gene significance for LM (MM vs. GS: *r* = 0.73, *P* = 8.3 × 10⁻⁴⁵; Fig. 5E). Consistently, the turquoise module eigengene increased progressively across clinical groups, peaking in LM+ samples (Fig. 5F), indicating a coherent LM-linked co-expression program.

In contrast, plasma networks showed weaker and less consistent associations with LM. The strongest plasma module demonstrated only moderate correlation with LM status, and overall module–phenotype coupling was attenuated compared with CSF (Supplementary Fig. S3A–E). Crucially, cross-biofluid analyses further indicated limited translatability of the CSF-derived LM signatures to the systemic circulation. The global eigengene correlation matrix revealed sparse and heterogeneous module correspondences between the two biofluids (Fig. 5G). Permutation-based preservation analysis confirmed this divergence, as evidenced by predominantly low Z*summary* values, indicating limited preservation of CSF modules in plasma and similarly weak preservation of plasma modules in CSF (Fig. 5H; Supplementary Fig. S3F). Consistently, GSVA-based module activity scores for the CSF turquoise and plasma blue modules were not correlated in paired samples (*R* = 0.20, *P* = 0.25; Fig. 5I). Together, these findings indicate that LM-associated sEV-related miRNA programs are strongly coordinated in CSF but are not reliably preserved in plasma, supporting the use of CSF-derived features for downstream biomarker development. Accordingly, hub miRNAs (MM > 0.8, GS > 0.5; Supplementary Fig. S3G) from the CSF LM-associated module were selected for subsequent model construction.

### Development of an sEV-associated miRNA diagnostic panel for LM

**Fig. 6:**
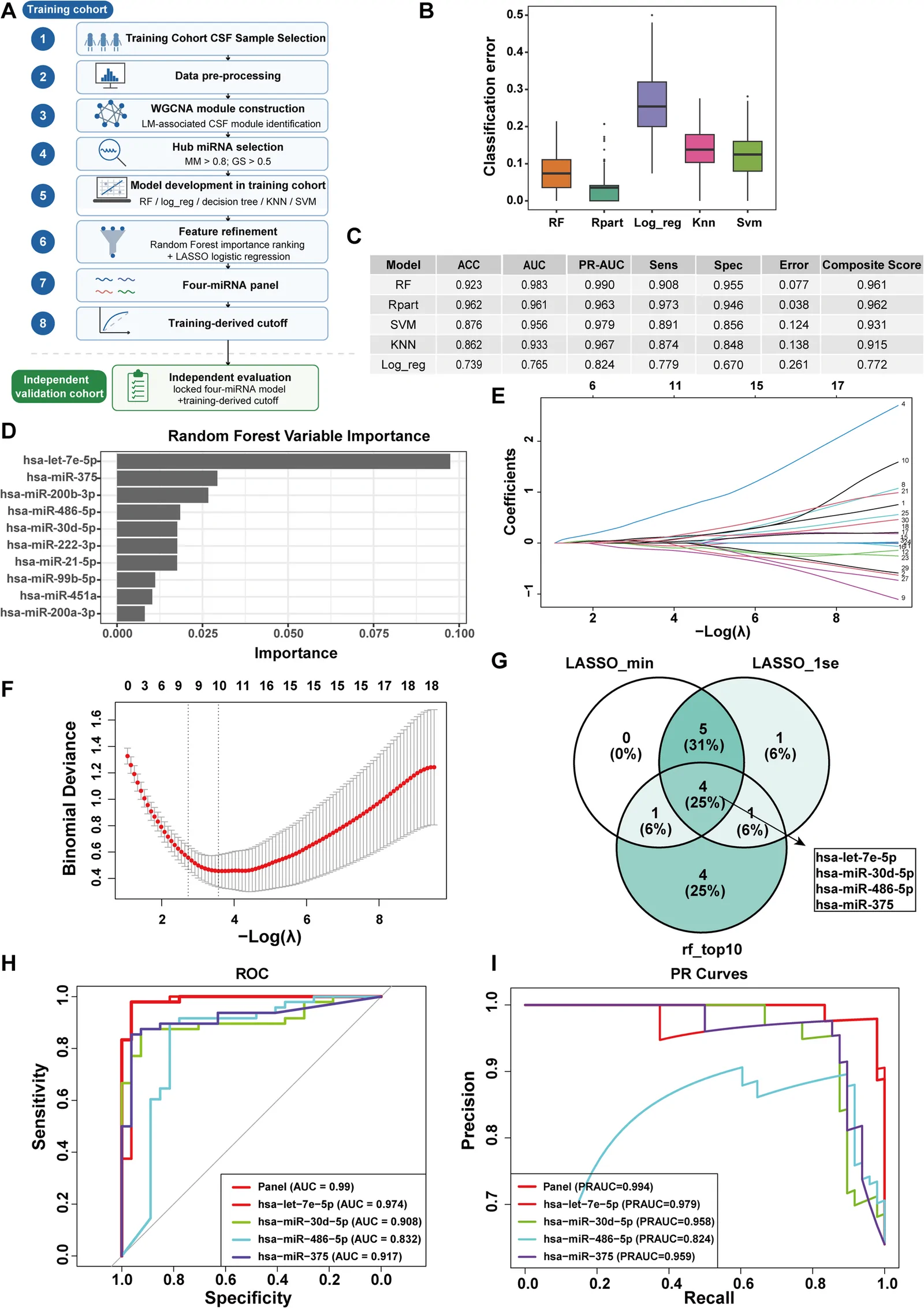
Machine learning–based development of a CSF sEV-associated miRNA diagnostic panel for LM. **(A)** Overview of the model development workflow. **(B)** Comparison of classification error rates across candidate machine learning models, including Random Forest (RF), Recursive Partitioning (Rpart), Logistic Regression (Log_reg), k-Nearest Neighbors (KNN), and Support Vector Machine (SVM). **(C)** Summary table of model performance metrics, including mean accuracy (ACC), area under the ROC curve (AUC), area under the precision–recall curve (PR-AUC), sensitivity (Sens), specificity (Spec), classification error (Error), and composite score. **(D)** Random Forest–based variable importance of candidate miRNAs estimated using permutation importance. The top 10 miRNAs contributing to classification performance are shown. **(E)** Coefficient profiles of miRNAs generated by LASSO regression as a function of − log(λ), showing feature shrinkage and selection paths. **(F)** Ten-fold cross-validation curve for LASSO regression. The binomial deviance is plotted against − log(λ), with dashed vertical lines indicating the optimal λ values (λ_min and λ_1se). **(G)** Venn diagram showing overlap among miRNAs selected by RF top 10 features, LASSO (λ_min), and LASSO (λ_1se). **(H)** ROC curves comparing the diagnostic performance of the combined sEV-associated miRNA panel and individual miRNAs, with corresponding AUC values indicated. **(I)** Precision–Recall curves of the sEV-associated miRNA panel and individual miRNAs

To develop a diagnostic signature for LM, we utilized CSF-derived sEV-associated miRNA profiles and constructed machine learning models based on candidate features identified from prior network analysis (Fig. 6A). Among the evaluated models, the classification-error distributions are shown in Fig. 6B, and Random Forest (RF) achieved the highest mean AUC and PR-AUC (Fig. 6C). Given its superior discriminative power, we next performed RF variable-importance ranking to identify the most informative miRNAs contributing to LM classification (Fig. 6D). By integrating permutation-based importance rankings with LASSO-based feature selection, we identified a four-miRNA diagnostic panel comprising hsa-let-7e-5p, hsa-miR-30d-5p, hsa-miR-486-5p, and hsa-miR-375 (Fig. 6E–G). We subsequently evaluated the diagnostic performance of this panel against individual miRNAs. The four-miRNA panel achieved an AUC of 0.99 and a PR-AUC of 0.994, markedly outperforming any single miRNA component (Fig. 6H, I). In comparison, individual miRNAs exhibited AUCs ranging from 0.832 to 0.974, with let-7e-5p and miR-375 showing the highest single-marker performance. Collectively, these results support the development of a compact CSF sEV-associated miRNA signature with strong discriminative capacity for LM.

As qualitative exploratory tissue-level context for the CSF-derived four-miRNA panel, we examined available matched FFPE specimens from one LM-positive patient. In this single matched case, miRNA FISH showed detectable signals for all four panel miRNAs in the primary NSCLC lesion, with more apparent signals for hsa-miR-375-3p and hsa-miR-486-5p. In contrast, the pathologically negative dural tissue showed minimal or absent probe signal. In the leptomeningeal metastatic tissue, hsa-miR-375, hsa-miR-30d-5p, and hsa-let-7e-5p were detectable to varying degrees, whereas hsa-miR-486-5p showed little or no apparent signal (Supplementary Fig. S4). These findings provide exploratory tissue-level spatial context for the CSF-derived four-miRNA panel.

### Diagnostic performance of the sEV-associated miRNA panel and comparison with conventional modalities

**Fig. 7:**
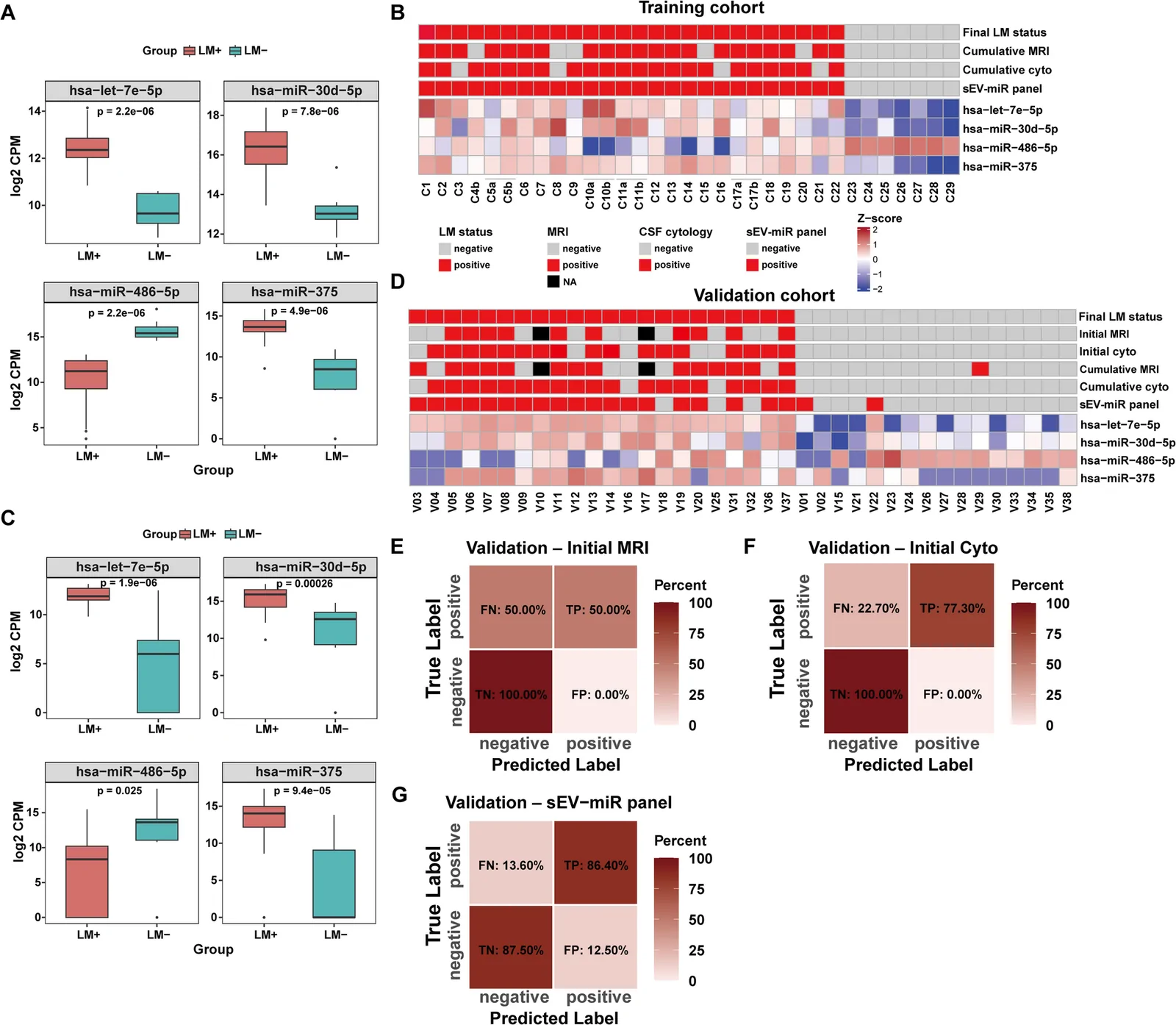
Diagnostic performance of the CSF sEV-associated miRNA panel compared with conventional clinical modalities. **(A)** Box-and-whisker plots showing differential expression of the four core miRNAs between LM-positive (LM+) and LM-negative (LM−) patients in the training cohort. **(B)** Patient-level heatmap in the training cohort summarizing final LM status, cumulative MRI, cumulative CSF cytology, and sEV-miRNA panel predictions, displayed alongside normalized expression Z-scores of the four-miRNA panel. **(C)** Box-and-whisker plots of the four core miRNAs in the validation cohort, demonstrating consistent expression patterns with the training cohort. **(D)** Patient-level heatmap in the validation cohort summarizing final LM status, initial MRI, initial CSF cytology, cumulative MRI, cumulative CSF cytology, and sEV-miRNA panel predictions. **(E)** Confusion matrix of initial MRI in the validation cohort. **(F)** Confusion matrix of initial CSF cytology in the validation cohort. **(G)** Confusion matrix of the sEV-miRNA panel in the validation cohort. Confusion matrices are normalized within each true diagnostic category. Final LM status was determined by clinical adjudication based on all available clinical information, including imaging, CSF cytology, biopsy when available, and longitudinal follow-up. The sEV-miRNA panel was not used for LM adjudication. Abbreviations: CSF, cerebrospinal fluid; LM, leptomeningeal metastasis; MRI, magnetic resonance imaging; sEV, small extracellular vesicle

To evaluate the translational potential of the four-miRNA signature, we assessed its diagnostic performance in both training and validation cohorts. In the training set, let-7e-5p, miR-30d-5p, and miR-375 were significantly upregulated in LM+ patients, while miR-486-5p was downregulated; these directional changes were independently recapitulated in the validation cohort (Fig. 7A, C), supporting the molecular robustness of the panel. To examine whether the four-miRNA pattern was evident at the individual-sample level, we further visualized the panel miRNAs in selected training-cohort samples (Supplementary Fig. S5). The expected CSF expression pattern was observed across non-cancer, LM−, and LM+ samples, with hsa-let-7e-5p, hsa-miR-30d-5p, and hsa-miR-375 increased and hsa-miR-486-5p decreased in LM + CSF samples (Supplementary Fig. S5A). Consistent with the global clustering result, C4 pre-LM CSF also showed a panel-like four-miRNA pattern similar to the matched post-LM sample, although this single-case observation remains hypothesis-generating (Supplementary Fig. S5B). Repeated CSF samples from C5, C10, C11, and C17 generally retained the panel-associated pattern during repeated sampling, although patient-specific temporal variability was observed, particularly for hsa-miR-486-5p. Plasma group-level patterns and matched pre/post trajectories were less consistent than the corresponding CSF patterns (Supplementary Fig. S5C–D), further supporting the compartment-restricted nature of the CSF-derived signature.

At the patient level, diagnostic performance was evaluated relative to final adjudicated LM status. In the training cohort, the panel correctly classified all evaluable LM + and LM− patients, corresponding to 100% sensitivity and specificity (Fig. 7B; Supplementary Table S3). In the independent validation cohort, the panel maintained strong discriminative capacity, achieving an AUC of 0.935 (Supplementary Table S4) and classifying 19 of 22 LM+ patients and 14 of 16 LM− patients correctly, corresponding to 86.4% sensitivity and 87.5% specificity (Fig. 7D, G; Supplementary Table S4). For contextual comparison with conventional diagnostic modalities, MRI and CSF cytology were analyzed as both initial and cumulative assessments. Initial assessments were defined as the first available test during evaluation for suspected LM, whereas cumulative assessments incorporated all available tests during clinical follow-up. In the validation cohort, initial MRI and initial CSF cytology showed sensitivities of 50.0% and 77.3%, respectively, compared with 86.4% for the single-sample sEV-miRNA panel (Fig. 7E–G; Supplementary Tables S2 and S4). Additional diagnostic parameters, including specificity, accuracy, PPV, and NPV, together with corresponding 95% confidence intervals, are provided in Supplementary Table S4.

Cumulative diagnostic yields of conventional modalities were also reported for clinical context. In the validation cohort, cumulative MRI and cumulative CSF cytology showed sensitivities of 70.0% and 86.4%, respectively (Supplementary Fig. S6D–E; Supplementary Table S4). Because cumulative assessments incorporated longitudinal clinical follow-up, these values were interpreted as cumulative diagnostic yields rather than single-time-point test-performance estimates. Together, these findings suggest that CSF sEV-associated miRNA profiling provides a single-sample molecular readout that may complement MRI and CSF cytology, particularly in clinically challenging cases with inconclusive imaging or negative initial cytology.

## Discussion

LM from NSCLC remains a major diagnostic bottleneck in neuro-oncology [5]. In routine practice, early LM evaluation often relies on contrast-enhanced MRI and CSF cytology. However, both approaches frequently yield inconclusive results at initial presentation, often necessitating repeated assessments and delaying treatment decisions [6]. In this context, a single-sample molecular assay that provides high-confidence information could meaningfully improve the diagnostic workflow. Although sEV-associated miRNA signatures have demonstrated clinical feasibility in large multicenter studies, such as the DESTINEX trial in gastric cancer [12, 13], their applicability to central nervous system (CNS) malignancies remains uncertain. As nanoscale carriers of protected molecular cargo, sEVs represent promising substrates for molecular diagnostics; however, in CNS-restricted diseases, their diagnostic fidelity is likely shaped by barrier effects such as the blood–brain barrier. This necessitates a compartment-aware sampling strategy. Accordingly, whether LM-associated sEV-miRNA signals are preserved across biofluids remains unclear [22].

In this study, through systematic profiling of sEV-associated miRNA landscapes in CSF and plasma from NSCLC patients with clinically adjudicated LM status, we demonstrate a pronounced compartmentalization of diagnostic information. CSF-derived sEV-associated miRNA profiles exhibited coherent LM-associated remodeling and robustly discriminated LM+ from LM− individuals. In contrast, plasma-derived signals were substantially attenuated and showed limited stratification capacity. This biofluid asymmetry supports the concept that, for malignancies confined behind barrier interfaces, proximal biofluids provide a substantially higher signal-to-noise ratio than systemic circulation. Mechanistically, the divergence between CSF and plasma is biologically plausible [18, 22]. CNS barrier systems, including the blood–brain and blood–CSF barriers, restrict the bidirectional exchange of molecular cargo, and the extent to which endogenous sEV traverse these interfaces remains incompletely understood [23]. In parallel, plasma contains a vast and heterogeneous sEV pool dominated by contributions from peripheral tissues and hematopoietic cells, as reflected by the predominance of hematopoietic-associated miRNAs (e.g., miR-223-3p) and endothelial-associated miRNAs (e.g., miR-126-3p) in our data. Consequently, this systemic background can overwhelm sparse CNS-derived vesicular signals and complicate biomarker discovery [17]. Consistent with these principles, our cross-fluid preservation analysis confirmed that the CSF-defined LM-associated miRNA program is not reliably mirrored in plasma. This finding has practical implications: plasma sEV-associated miRNA measurements should not be assumed to serve as a faithful proxy for CSF-derived molecular remodeling in LM. More broadly, these results support a compartment-aware framework for extracellular vesicle-based biomarker development in CNS-restricted diseases.

We acknowledge that sEV isolation methods, including ultracentrifugation and SEC, do not yield pure sEV populations, and that the RNA cargo analyzed in this study should therefore be interpreted as originating from sEV-enriched fractions rather than exclusively sEV-bound molecules [19, 24, 25]. RNA extraction was performed using the same procedure for both CSF- and plasma-derived sEV-enriched fractions, reducing extraction-related variability. However, different upstream isolation strategies were applied to CSF and plasma because of biofluid-specific constraints. Plasma contains abundant soluble proteins, lipoproteins, and other non-vesicular particles; therefore, SEC was used to reduce protein-rich contaminants. In contrast, CSF is characterized by a lower protein background but also lower vesicle and RNA abundance, and ultracentrifugation was used to maximize vesicle recovery from limited CSF samples [25].

We recognize that biofluid-specific isolation workflows may contribute to quantitative and compositional differences between CSF and plasma sEV-miRNA profiles. Accordingly, cross-biofluid comparisons should be interpreted with caution. However, the main four-miRNA diagnostic panel was developed and validated entirely within CSF samples processed using a consistent CSF workflow. Therefore, its training-to-validation performance is not affected by differences between CSF and plasma isolation workflows. Moreover, the observed CSF–plasma divergence is biologically plausible and is unlikely to be explained solely by technical factors. A recent integrative single-cell and spatial profiling study of leptomeningeal disease showed that CSF can function as an immune-active and disease-informative compartment, with disease-specific cellular landscapes including T-cell activation/exhaustion and clonal T-cell expansion in CNS lymphoma-associated leptomeningeal disease, and myeloid-dominant profiles in brain metastasis- and glioblastoma-associated leptomeningeal disease. In that study, matched spatial transcriptomic analyses further showed that CSF immune phenotypes can, in some cases, mirror key features of CNS lesion microenvironments, supporting the concept that CSF provides a proximal readout of CNS disease biology rather than simply reflecting systemic circulation [26]. In this context, the failure of plasma to recapitulate our CSF-derived miRNA module is consistent with biological compartmentalization, although method-related effects cannot be completely excluded.

A notable observation in our longitudinal analysis was a potential molecular lead time, in which a pre-diagnostic CSF sample clustered with its corresponding post-diagnosis sample. Because this finding derives from a single case, it should be viewed as hypothesis-generating rather than evidence of established early-detection capability. Nevertheless, it raises the possibility that CSF sEV-associated miRNA alterations may precede radiologic or cytologic confirmation in selected patients. Given the known diagnostic challenges of MRI and cytology, especially at early stages, this hypothesis warrants dedicated validation in larger longitudinal and high-risk cohorts [6]. Reinforcing this case-level observation, our WGCNA framework identified a dominant CSF turquoise module tightly coupled to LM status, suggesting that CSF sEV-associated miRNAs may capture a coordinated LM-associated molecular program rather than merely reflecting random molecular noise.

By leveraging biologically informed features from the CSF turquoise module, we distilled high-dimensional sequencing data into a parsimonious four-miRNA panel comprising hsa-let-7e-5p, hsa-miR-30d-5p, hsa-miR-486-5p, and hsa-miR-375. These miRNAs should not be interpreted as isolated biomarkers, but rather as components of a broader CSF LM-associated co-expression program. The biological themes potentially reflected by this panel include epithelial plasticity, vesicle-mediated intercellular communication, barrier remodeling, EMT-associated signaling, growth-factor pathway regulation, and tumor-associated molecular release into the CSF. Among the four miRNAs, hsa-miR-375 has the most direct experimental link to barrier disruption. Extracellular vesicle-associated miR-375 has been shown to impair endothelial tight-junction integrity by downregulating claudin-1, thereby increasing vascular permeability and promoting transendothelial tumor-cell migration. In vivo, miR-375-enriched exosomes induced vascular permeability changes in multiple organs, including the brain [27]. Although these findings were demonstrated in SCLC rather than NSCLC-associated LM, they provide an indirect but biologically plausible mechanistic context linking EV-associated miR-375 to barrier compromise relevant to CNS or leptomeningeal dissemination. The remaining three miRNAs provide less direct but complementary biological context for the CSF LM-associated program. hsa-let-7e-5p belongs to the let-7 family, which has been broadly implicated in lung cancer biology, epithelial differentiation, and metastatic regulation. Serum-derived exosomal miR-let-7e has been reported to suppress NSCLC cell migration and invasion through the SUV39H2/LSD1/CDH1 axis, providing indirect support for a link between vesicle-associated let-7e and epithelial-state regulation [28]. hsa-miR-30d-5p has been linked to TGF-β1-associated regulation of EMT-related genes in A549 lung cancer cells, including DUSP-1, E-cadherin, and Vimentin, providing indirect support for a connection between miR-30d-5p and epithelial plasticity or EMT-associated remodeling [29]. By contrast, hsa-miR-486-5p has been reported to act as a tumor-suppressive miRNA in NSCLC models, inhibiting proliferation and migration, downregulating mTOR signaling, and attenuating EMT-associated phenotypes [30]. Beyond their EMT-associated effects, available studies have also linked miR-30d-5p to immune-regulatory signaling and miR-486-5p to cisplatin sensitivity in NSCLC models; however, these observations were established outside the CSF and LM settings [31, 32]. The reduced abundance of hsa-miR-486-5p in LM+ CSF is directionally consistent with attenuation of tumor-suppressive miRNA-associated programs, although extracellular CSF abundance cannot be directly equated with intracellular miRNA activity.

Taken together, these findings provide biologically plausible context for the individual panel miRNAs, but they should not be interpreted as direct evidence of their roles in NSCLC-associated LM. Most supporting studies were conducted in tumor tissues, blood-derived EVs, or experimental lung cancer models rather than in CSF from patients with LM, and direct evidence linking hsa-let-7e-5p, hsa-miR-30d-5p, or hsa-miR-486-5p to CNS or leptomeningeal metastasis remains limited. Several panel members, including hsa-miR-486-5p, hsa-miR-30d-5p, and hsa-let-7e-5p, have also been detected in human CSF outside the LM setting, indicating that their presence alone is not LM-specific [33]. More broadly, CSF and CSF-EV miRNA profiles can be altered in primary CNS tumors, metastatic brain disease [34], neurological injury [35], neurodegenerative disorders [36], hydrocephalus [37], and age-associated biological states [38]. However, the coordinated directionality of the complete four-miRNA signature has not been systematically evaluated across these alternative conditions or in treatment-related neurological complications. Our findings therefore support the panel as a CSF compartment-associated diagnostic signature rather than proof of an LM-specific biological mechanism, and its disease specificity should be further assessed in broader CSF disease-control cohorts.

Beyond these disease-specificity considerations, the cellular origin and biological meaning of the CSF four-miRNA signature remain incompletely resolved. Because matched leptomeningeal or CNS metastatic tissue suitable for miRNA profiling was not available for most patients, and CSF cfDNA-based tumor fraction data were not incorporated, we cannot determine whether the observed CSF sEV-miRNA changes primarily reflect tumor-derived vesicle shedding, selective miRNA cargo sorting, local leptomeningeal microenvironmental remodeling, or a combination of these processes. Nevertheless, several observations argue against a purely nonspecific shedding model. The CSF sEV-miRNA profiles separated LM+ patients from LM− controls despite the high prevalence of intraparenchymal brain metastases among LM− controls, and the LM-associated miRNA program was prominent in CSF but not recapitulated in plasma. Exploratory single-case tissue FISH provided additional spatial context by showing variable detectability of the four panel miRNAs across available primary NSCLC, pathologically negative dural, and leptomeningeal metastatic tissues. However, this analysis does not establish the cellular or vesicular origin of CSF sEV-miRNAs, because it was based on a single matched case and tissue FISH cannot trace EV release into CSF. Therefore, the four-miRNA panel is best interpreted as an LM-proximal, compartment-restricted CSF sEV-miRNA diagnostic signature that may capture a combination of tumor-derived vesicle shedding, selective cargo sorting, local leptomeningeal microenvironmental remodeling, and other LM-relevant biological processes.

From a clinical perspective, such a compartment-restricted single-sample molecular readout is particularly relevant because LM diagnosis in routine clinical practice remains challenging and typically relies on an integrated assessment of neurological symptoms, contrast-enhanced MRI, CSF cytology, and longitudinal clinical course. When initial imaging or cytology is inconclusive, follow-up imaging, repeated CSF cytology, and, in selected cases, meningeal biopsy may contribute to final clinical adjudication. Consistent with this diagnostic complexity, a recent systematic review and meta-analysis reported pooled sensitivities at initial diagnosis of 69.4% for MRI and 71.9% for CSF cytology in solid cancers, underscoring the limitations of single time-point testing [6].

Our validation cohort further highlights the difference between single time-point testing and longitudinal clinical assessment in LM diagnosis. Initial MRI and initial CSF cytology showed sensitivities of 50.0% and 77.3%, respectively. With follow-up imaging and repeated CSF cytology incorporated, cumulative sensitivities increased to 70.0% for MRI and 86.4% for CSF cytology. These findings are consistent with the clinical reality that LM diagnosis often requires repeated or longitudinal assessment when initial imaging or cytology is inconclusive. By contrast, the sEV-miRNA panel achieved 86.4% sensitivity and 87.5% specificity from a single CSF sample—a sensitivity comparable to that of cumulative CSF cytology but without the need for repeated lumbar punctures. Because MRI and CSF cytology contributed to the final adjudicated LM status, these comparisons should be interpreted as diagnostic yields relative to clinical adjudication rather than fully independent head-to-head test-performance estimates. Within this framework, the sEV-miRNA panel may serve as a complementary single-sample molecular readout alongside conventional diagnostic modalities.

### Limitations and future directions

Several limitations warrant consideration. First, cohort size was constrained by the rarity of NSCLC-associated LM and the invasiveness of CSF acquisition, highlighting the need for larger, multicenter prospective studies to confirm diagnostic performance across diverse populations and clinical settings. Second, treatment regimens and molecular subtypes, such as EGFR-mutant versus non–EGFR-mutant disease, may influence sEV composition and miRNA cargo. However, because LM typically arises as a late-stage complication, most patients have undergone extensive and heterogeneous therapeutic interventions by the time of diagnosis, making it challenging to perform granular stratified analyses based on prior treatment trajectories. Future studies with larger and more uniform cohorts should account for treatment history and tumor genomic context. Third, the cellular and anatomical origin of the four-miRNA CSF signature could not be definitively resolved. Pathologically confirmed leptomeningeal or CNS metastatic tissues suitable for miRNA profiling were scarce because meningeal biopsy is rarely performed in routine clinical practice. Exploratory miRNA FISH in one LM-positive patient provided preliminary tissue-level spatial context but could not establish the vesicular or cellular origin of the CSF signals. In addition, CSF cfDNA-based tumor fraction, CSF tumor-cell burden, and tumor-origin EV markers were not assessed. Future studies integrating these measurements with clinically available tumor tissues will be required to distinguish tumor-derived vesicle release from local leptomeningeal microenvironmental remodeling. Fourth, although the CSF four-miRNA panel was developed and validated using a consistent CSF workflow, broader validation is required to establish robustness across sites, sEV isolation workflows, and assay platforms. Orthogonal validation using targeted qRT-PCR or ddPCR was not performed because CSF samples from patients with suspected LM are limited in volume, and must prioritize routine diagnostic testing, including cytology and biochemical analyses. In the present cohort, the available research CSF was used for sEV enrichment and small RNA-seq library preparation, leaving insufficient residual CSF-derived sEV RNA for reliable targeted validation with appropriate controls and technical replicates. Future prospective studies should therefore predefine CSF allocation for both sequencing-based discovery and orthogonal targeted validation using qRT-PCR, ddPCR, or other standardized assays. Inter-laboratory reproducibility assessments will also be essential for clinical translation. Finally, the molecular lead time observation remains a proof-of-concept derived from a single patient and should be evaluated in longitudinal screening cohorts of high-risk individuals.

## Conclusion

In summary, this study demonstrates that CSF-derived sEV-associated miRNAs capture a coherent LM-proximal molecular signal that is largely absent from plasma, and that exploiting this compartment-specific signal enables a compact diagnostic panel with promising diagnostic performance. More broadly, our findings support a compartment-aware paradigm for EV biomarker development in CNS malignancies: when disease biology is sequestered behind barrier interfaces, proximal biofluids may provide a more disease-informative molecular readout, whereas systemic circulation may offer convenience at the cost of diluted fidelity. With further validation and clinical integration, CSF sEV-associated miRNA profiling may complement conventional diagnostics, reduce reliance on repeated procedures, and support more timely and reliable LM evaluation in NSCLC.

## Supplementary Information

Supplementary Material

## Data Availability

The raw data generated in this study are available from the corresponding author on reasonable request.

## Abbreviations

LM: Leptomeningeal metastasis
NSCLC: Non-small cell lung cancer
EGFR: Epidermal growth factor receptor
MRI: Magnetic resonance imaging
CSF: Cerebrospinal fluid
sEV: Small extracellular vesicle
miRNA: MicroRNA
CNS: Central nervous system
BBB: Blood–brain barrier
CTCs: Circulating tumor cells
ctDNA: Circulating tumor DNA
IM: Intraparenchymal brain metastasis
WGCNA: Weighted gene co-expression network analysis
SEC: Size-exclusion chromatography
NTA: Nanoparticle tracking analysis
TEM: Transmission electron microscopy
LTF: Leptomeningeal tumor-free
PCA: Principal component analysis
DE: Differential expression
GO: Gene ontology
KEGG: Kyoto encyclopedia of genes and genomes
RF: Random forest
LASSO: Least absolute shrinkage and selection operator
