# Supplementary material for: Cerebrospinal fluid-derived small extracellular vesicles reveal a compartment-specific diagnostic signature for NSCLC leptomeningeal metastasis
Source: J Nanobiotechnology. 2026 Aug 12;24:1050. doi: 10.1186/s12951-026-04847-8 (PMC13749793; doi:10.1186/s12951-026-04847-8)
Supplement: Supplementary file 1 [file 12951_2026_4847_MOESM2_ESM.docx]

**Cerebrospinal Fluid-Derived Small Extracellular Vesicles Reveal a Compartment-Specific Diagnostic Signature for NSCLC Leptomeningeal Metastasis**

**Authors:**

Peng-peng Kuang^12†^, Yang-si Li^1†^, Ting Hu^3†^, Mei-mei Zheng^1^, Jia-tao Zhang^1^, Kai Yin^12^, Jin-ji Yang ^1^, Xue-ning Yang ^1^, Qing Zhou ^1^, Hai-yan Tu ^1^, Yi-long Wu ^1^, Wen-zhao Zhong^12^**^*^**, Ben-yuan Jiang^1^**^*^**

**Affiliations:**

^1^Guangdong Lung Cancer Institute, Guangdong Cardiovascular Institute, Guangdong Provincial People’s Hospital, Guangdong Academy of Medical Sciences, Southern Medical University, Guangzhou, China.

^2^Guangdong Provincial Key Laboratory of Translational Medicine in Lung Cancer, Guangdong Provincial People’s Hospital, Guangdong Academy of Medical Sciences, Southern Medical University, Guangzhou, China.

^3^Department of Endoscopy, State Key Laboratory of Oncology in South China, Guangdong Provincial Clinical Research Center for Cancer, Sun Yat-Sen University Cancer Center, Guangzhou, China.

^†^ These authors contributed equally to this work.

***Corresponding author:**

Ben-yuan Jiang, Guangdong Lung Cancer Institute, Guangdong Provincial People’s Hospital, Guangdong Academy of Medical Sciences, Southern Medical University, 106 Zhongshan Er Rd, Guangzhou 510080, China

E-mail: [jiangben1000@126.com](mailto:jiangben1000@126.com);

Wen-zhao Zhong, Guangdong Lung Cancer Institute, Guangdong Provincial People’s Hospital, Guangdong Academy of Medical Sciences, Southern Medical University, 106 Zhongshan Er Rd, Guangzhou 510080, China

E-mail: syzhongwenzhao@scut.edu.cn

**Supplementary Figures**

**Supplementary Fig. S1**. **Subgroup differential expression analyses and functional enrichment of targets associated with upregulated sEV-associated miRNAs.** **(A–C)** Volcano plots for CSF subgroup contrasts: **(A)** LM− vs non-cancer, **(B)** LM+ vs non-cancer, and **(C)** LM+ vs LM−. **(D)** KEGG pathway enrichment of predicted target genes associated with upregulated differentially expressed sEV-associated miRNAs. Bubble size represents gene counts, and color indicates significance (−log10 FDR). **(E–G)** Gene Ontology (GO) enrichment analysis of predicted targets of upregulated differentially expressed sEV-associated miRNAs, shown for Molecular Function **(E)**, Cellular Component **(F)**, and Biological Process **(G)**. Bubble size indicates gene counts, and color indicates significance (−log10 FDR).

**Supplementary Fig. S2. Differential expression and functional enrichment analysis of sEV-associated miRNAs between matched CSF and plasma. (A)** Principal component analysis (PCA) of sEV-associated miRNA expression profiles from paired CSF and plasma samples (n = 22 pairs). **(B)** Volcano plot showing paired differential expression between CSF and plasma. Significantly upregulated and downregulated miRNAs (FDR < 0.05 and |log2FC| > 1) are highlighted. **(C)** Heatmap of the top 20 upregulated and top 20 downregulated sEV-associated miRNAs ranked by FDR. **(D–G)** Functional enrichment analysis of predicted target genes associated with plasma-enriched sEV-associated miRNAs, including **(D)** KEGG pathways and **(E–G)** Gene Ontology (GO) categories: Molecular Function **(E)**, Cellular Component **(F)**, and Biological Process **(G)**. **(H–K)** Functional enrichment analysis of predicted target genes associated with CSF-enriched sEV-associated miRNAs, including **(H)** KEGG pathways and **(I–K)** Gene Ontology (GO) categories: Molecular Function **(I)**, Cellular Component **(J)**, and Biological Process **(K)**. For bubble plots, dot size represents gene counts and color indicates significance (−log10 FDR).

**Supplementary Fig. S3**. **Weighted gene co-expression network analysis (WGCNA) of plasma sEV-associated miRNAs and cross-biofluid comparison. (A)** Sample clustering dendrogram of plasma samples. **(B)** Soft-thresholding analysis identifies power = 5 as the selected parameter for network construction. **(C)** Clustering dendrogram and module assignment illustrating discrete plasma sEV-associated miRNAs co-expression modules. **(D)** Module–trait correlation heatmap showing associations between plasma module eigengenes and LM status. **(E)** Scatter plot of module membership (MM) versus gene significance (GS) for miRNAs in the plasma blue module. **(F)** Module preservation analysis using plasma as the reference dataset to assess preservation in CSF. Zsummary thresholds indicate strong preservation (Z > 10), moderate preservation (2 < Z < 10), or no/weak preservation (Z < 2). **(G)** Scatter plot of module membership (MM) versus gene significance (GS) for miRNAs in the CSF turquoise module (from Fig. 5), with hub-selection thresholds (MM > 0.8, GS > 0.5) indicated to define the high-confidence hub subset.

**Supplementary Fig. S4. Exploratory tissue-level spatial context for the four-miRNA CSF signature.** Representative miRNA FISH images of available matched FFPE specimens from one LM-positive patient, including a primary NSCLC lesion, pathologically negative dural tissue, and leptomeningeal metastatic tissue. Columns show FISH staining for hsa-miR-375, hsa-miR-486-5p, hsa-miR-30d-5p, and hsa-let-7e-5p. hsa-miR-375 and hsa-miR-30d-5p were detected using Cy3-labelled probes, hsa-miR-486-5p using a Cy5-labelled probe, and hsa-let-7e-5p using a FAM-labelled probe. Nuclei were counterstained with DAPI. Scale bars, 25 μm.

**Supplementary Fig. S5. Individual-level CSF and plasma expression patterns of the four sEV-miRNA panel components in selected training-cohort samples.
(A)** CSF expression levels of hsa-let-7e-5p, hsa-miR-30d-5p, hsa-miR-486-5p, and hsa-miR-375 across non-cancer, LM−, and LM+ samples in the training cohort. **(B)** Individual CSF trajectories of the four panel miRNAs in selected paired or repeated samples, including the pre- and post-LM CSF samples from patient C4 and sequential CSF samples from patients C5, C10, C11, and C17. **(C)** Plasma expression levels of the four panel miRNAs across healthy individuals, non-cancer controls, LM− patients, pre-LM+ samples, and post-LM+ samples in the training cohort. **(D)** Matched plasma pre- and post-LM trajectories of the four panel miRNAs in selected LM+ patients. Expression values are shown as log2(CPM + 1). Adjusted P values are shown for the indicated comparisons. Healthy individuals were included only in the plasma analysis because CSF collection from neurologically healthy volunteers solely for research purposes was not ethically justified.

**Supplementary Fig. S6. Confusion matrices for cumulative conventional diagnostic modalities and the CSF sEV-miRNA panel.** (A) Confusion matrix for cumulative MRI in the training cohort. (B) Confusion matrix for cumulative CSF cytology in the training cohort. (C) Confusion matrix for the CSF sEV-miRNA panel in the training cohort. (D) Confusion matrix for cumulative MRI in the validation cohort. (E) Confusion matrix for cumulative CSF cytology in the validation cohort. True labels indicate final adjudicated LM status, and predicted labels indicate the corresponding test results. Cumulative MRI and cumulative CSF cytology were considered positive if any available assessment during clinical follow-up was positive. All confusion matrices are normalized to percentages within each true diagnostic category, thereby illustrating patient-level sensitivity and specificity. FN, false negative; FP, false positive; TN, true negative; TP, true positive; LM, leptomeningeal metastasis; MRI, magnetic resonance imaging; CSF, cerebrospinal fluid; sEV, small extracellular vesicle.

**Supplementary tables**

**Supplementary Table S1. Clinical characteristics and biospecimen collection schema of the training cohort**

| **Patient** | **Age** | **Sex** | **Histology** | **Stage** | **Cumulative MRI** | **Cumulative Cyto** | **Final LM**  **status** | **IM**  **status** | **CSF collected** | | **Plasma collected** | |
| --- | --- | --- | --- | --- | --- | --- | --- | --- | --- | --- | --- | --- |
| C1 | 65 | F | AC | IV | pos | pos | LM+ | IM- |  | post | pre | post |
| C2 | 35 | F | AC | IV | pos | pos | LM+ | IM+ |  | post | pre | post |
| C3 | 59 | M | AC | IV | pos | neg | LM+ | IM+ |  | post | pre | post |
| C4 | 62 | F | AC | IV | neg | pos | LM+ | IM+ | pre | post | pre | post |
| C5 | 65 | M | AC | IV | pos | pos | LM+ | IM+ | post(repeat) | post | pre | post |
| C6 | 69 | F | AC | IV | pos | pos | LM+ | IM+ |  | post | pre | post |
| C7 | 56 | F | AC | IV | pos | pos | LM+ | IM- |  | post | pre | post |
| C8 | 42 | F | AC | IV | neg | neg | LM+ | IM+ |  | post | pre | post |
| C9 | 53 | F | AC | IV | neg | pos | LM+ | IM- |  | post | pre | post |
| C10 | 58 | F | AC | IV | pos | pos | LM+ | IM- | post(repeat) | post | pre | post |
| C11 | 60 | F | AC | IV | pos | pos | LM+ | IM+ | post(repeat) | post | pre | post |
| C12 | 35 | F | AC | IV | pos | pos | LM+ | IM+ |  | post | pre | post |
| C13 | 36 | F | AC | IV | pos | pos | LM+ | IM+ |  | post | pre | post |
| C14 | 61 | M | AC | IV | pos | pos | LM+ | IM- |  | post | pre | post |
| C15 | 42 | M | AC | IV | neg | pos | LM+ | IM+ |  | post | pre | post |
| C16 | 76 | F | AC | IV | pos | neg | LM+ | IM+ |  | post | pre | post |
| C17 | 45 | F | AC | IV | pos | pos | LM+ | IM+ | post(repeat) | post | pre | post |
| C18 | 62 | F | AC | IV | pos | pos | LM+ | IM+ |  | post | pre | post |
| C19 | 60 | M | AC | IV | pos | pos | LM+ | IM+ |  | post | pre | post |
| C20 | 43 | F | AC | IV | neg | pos | LM+ | IM- |  | post | pre | post |
| C21 | 59 | F | AC | IV | pos | neg | LM+ | IM- |  | post | pre | post |
| C22 | 37 | M | AC | IV | pos | pos | LM+ | IM+ |  | post | pre | post |
| C23 | 57 | M | AC | IV | neg | neg | LM- | IM+ |  | post |  | post |
| C24 | 59 | F | AC | IV | neg | neg | LM- | IM+ |  | post |  | post |
| C25 | 69 | F | AC | IV | neg | neg | LM- | IM+ |  | post |  | post |
| C26 | 73 | M | AC | IV | neg | neg | LM- | IM+ |  | post |  | post |
| C27 | 47 | M | AC | IV | neg | neg | LM- | IM+ |  | post |  | post |
| C28 | 57 | F | AC | IV | neg | neg | LM- | IM- |  | post |  | post |
| C29 | 57 | F | AC | IV | neg | neg | LM- | IM+ |  | post |  | post |
| C40 | 26 | M | Non-cancer | NA | NA | NA | NA | NA | At enrollment | | At enrollment | |
| C41 | 68 | M | Non-cancer | NA | NA | NA | NA | NA | At enrollment | | At enrollment | |
| C42 | 48 | F | Non-cancer | NA | NA | NA | NA | NA | At enrollment | | At enrollment | |
| C43 | 52 | M | Non-cancer | NA | NA | NA | NA | NA | At enrollment | | At enrollment | |
| C44 | 44 | F | Healthy | NA | NA | NA | NA | NA |  | | At enrollment | |
| C45 | 31 | F | Healthy | NA | NA | NA | NA | NA |  | | At enrollment | |
| C46 | 47 | M | Healthy | NA | NA | NA | NA | NA |  | | At enrollment | |
| C47 | 40 | F | Healthy | NA | NA | NA | NA | NA |  | | At enrollment | |
| C48 | 30 | F | Healthy | NA | NA | NA | NA | NA |  | | At enrollment | |
| C49 | 29 | M | Healthy | NA | NA | NA | NA | NA |  | | At enrollment | |
| C50 | 38 | F | Healthy | NA | NA | NA | NA | NA |  | | At enrollment | |
| C51 | 33 | F | Healthy | NA | NA | NA | NA | NA |  | | At enrollment | |

**Note:** Cumulative MRI and Cumulative Cyto refer to the results of all available MRI or CSF cytology assessments during clinical follow-up; patients were annotated as positive if any available assessment was positive. Final LM indicates the final adjudicated LM status based on MRI, CSF cytology, meningeal biopsy when available, and longitudinal follow-up. “Pre” and “Post” indicate samples collected before and after the clinical diagnosis of LM, respectively. “Post (repeat)” indicates additional post-diagnosis CSF samples obtained via repeat lumbar puncture. Missing values indicate that samples were not available for collection. **Abbreviations:** NSCLC, non-small cell lung cancer; LM, leptomeningeal metastasis; IM, intraparenchymal metastasis; CSF, cerebrospinal fluid; MRI, magnetic resonance imaging; AC, adenocarcinoma; IV, stage IV; pos, positive; neg, negative; F, female; M, male.

**Supplementary Table S2. Clinical characteristics and biospecimen collection scheme of the validation cohort.**

| **Patient** | **Age** | **Sex** | **Histology** | **Stage** | **Initial MRI** | **Initial Cyto** | **Cumulative MRI** | **Cumulative Cyto** | **sEV-miR panel** | **Final LM**  **status** | **IM**  **status** | **CSF collected** | | **Plasma collected** | |
| --- | --- | --- | --- | --- | --- | --- | --- | --- | --- | --- | --- | --- | --- | --- | --- |
| V01 | 65 | F | AC | IV | neg | neg | neg | neg | pos | LM- | IM- |  | post |  |  |
| V02 | 55 | F | AC | IV | neg | neg | neg | neg | neg | LM- | IM- |  | post |  |  |
| V03 | 36 | F | AC | IV | neg | neg | pos | neg | pos | LM+ | IM- |  | post |  |  |
| V04 | 56 | F | AC | IV | neg | pos | neg | pos | pos | LM+ | IM+ |  | post |  | post |
| V05 | 36 | M | AC | IV | pos | pos | pos | pos | pos | LM+ | IM- |  | post |  | post |
| V06 | 49 | F | AC | IV | pos | pos | pos | pos | pos | LM+ | IM- |  | post |  | post |
| V07 | 54 | M | AC | IV | pos | pos | pos | pos | pos | LM+ | IM+ |  | post |  | post |
| V08 | 41 | F | AC | IV | pos | pos | pos | pos | pos | LM+ | IM- |  | post |  | post |
| V09 | 40 | F | ASC | IV | neg | pos | neg | pos | pos | LM+ | IM+ |  | post |  |  |
| V10 | 65 | F | AC | IV | NA | pos | NA | pos | pos | LM+ | IM+ |  | post |  | post |
| V11 | 55 | M | AC | IV | pos | pos | pos | pos | pos | LM+ | IM+ |  | post |  | post |
| V12 | 61 | F | AC | IV | neg | neg | pos | pos | pos | LM+ | IM- |  | post |  | post |
| V13 | 56 | M | AC | IV | pos | pos | pos | pos | pos | LM+ | IM+ |  | post |  | post |
| V14 | 72 | M | AC | IV | neg | pos | neg | pos | pos | LM+ | IM+ |  | post |  | post |
| V15 | 63 | F | AC | IV | neg | neg | neg | neg | neg | LM- | IM+ |  | post |  |  |
| V16 | 62 | M | AC | IV | neg | neg | neg | neg | pos | LM+ | IM- |  | post |  |  |
| V17 | 56 | M | AC | IV | NA | pos | NA | pos | pos | LM+ | IM+ |  | post |  | post |
| V18 | 55 | F | AC | IV | neg | pos | neg | pos | neg | LM+ | IM+ |  | post |  | post |
| V19 | 35 | F | AC | IV | pos | pos | pos | pos | pos | LM+ | IM+ |  | post |  | post |
| V20 | 57 | F | AC | IV | pos | neg | pos | pos | pos | LM+ | IM+ |  | post |  | post |
| V21 | 69 | F | AC | IV | neg | neg | neg | neg | neg | LM- | IM+ |  | post |  |  |
| V22 | 54 | M | AC | IV | neg | neg | neg | neg | pos | LM- | IM+ |  | post |  |  |
| V23 | 59 | F | AC | IV | neg | neg | neg | neg | neg | LM- | IM+ |  | post |  |  |
| V24 | 75 | F | AC | IV | neg | neg | neg | neg | neg | LM- | IM+ |  | post |  |  |
| V25 | 59 | F | AC | IV | neg | neg | pos | neg | neg | LM+ | IM+ |  | post |  |  |
| V26 | 64 | M | AC | IV | neg | neg | neg | neg | neg | LM- | IM+ |  | post |  |  |
| V27 | 66 | M | AC | IV | neg | neg | neg | neg | neg | LM- | IM+ |  | post |  |  |
| V28 | 37 | M | AC | IV | neg | neg | neg | neg | neg | LM- | IM+ |  | post |  |  |
| V29 | 60 | M | AC | IV | neg | neg | pos | neg | neg | LM- | IM- |  | post |  |  |
| V30 | 48 | F | AC | IV | neg | neg | neg | neg | neg | LM- | IM+ |  | post |  |  |
| V31 | 42 | F | AC | IV | pos | pos | pos | pos | pos | LM+ | IM- |  | post |  | post |
| V32 | 63 | F | AC | IV | neg | pos | pos | pos | neg | LM+ | IM+ |  | post |  | post |
| V33 | 65 | F | AC | IV | neg | neg | neg | neg | neg | LM- | IM+ |  | post |  |  |
| V34 | 70 | F | AC | IV | neg | neg | neg | neg | neg | LM- | IM+ |  | post |  |  |
| V35 | 71 | F | AC | IV | neg | neg | neg | neg | neg | LM- | IM+ |  | post |  |  |
| V36 | 54 | F | AC | IV | neg | pos | neg | pos | pos | LM+ | IM+ |  | post |  |  |
| V37 | 57 | M | AC | IV | pos | pos | pos | pos | pos | LM+ | IM- |  | post |  | post |
| V38 | 55 | F | AC | IV | neg | neg | neg | neg | neg | LM- | IM+ |  | post |  |  |

**Note:** Initial MRI and Initial Cyto refer to the first available MRI and CSF cytology results during clinical LM evaluation for suspected LM. Cumulative MRI and Cumulative Cyto refer to the results of all available MRI or CSF cytology assessments during clinical follow-up; patients were annotated as positive if any available assessment was positive. Final LM indicates the final adjudicated LM status based on MRI, CSF cytology, meningeal biopsy when available, and longitudinal follow-up. The sEV-miRNA panel was not used for LM adjudication. All CSF samples in the validation cohort were collected after clinical LM evaluation and are annotated as “post”. Plasma samples were collected when available and are annotated as “post”. NA indicates that the corresponding test result was not available, whereas blank entries indicate that samples were not collected. **Abbreviations:** NSCLC, non-small cell lung cancer; LM, leptomeningeal metastasis; IM, intraparenchymal metastasis; CSF, cerebrospinal fluid; MRI, magnetic resonance imaging; AC, adenocarcinoma; ASC, adenosquamous carcinoma; IV, stage IV; pos, positive; neg, negative; NA, not available; F, female; M, male

**Supplementary Table S3. Diagnostic yield of cumulative conventional assessments and the single-sample sEV-miRNA panel relative to final adjudicated LM status in the training cohort.**

| **Diagnostic method** | **AUC**  **(95% CI)** | **Sensitivity, %**  **(n/N; 95% CI)** | **Specificity, %**  **(n/N; 95% CI)** | **Accuracy, %**  **(n/N; 95% CI)** | **PPV, %**  **(n/N; 95% CI)** | **NPV, %**  **(n/N; 95% CI)** |
| --- | --- | --- | --- | --- | --- | --- |
| Cumulative MRI | NA | 77.3  (17/22; 56.6–89.9) | 100.0  (7/7; 64.6–100.0) | 82.8  (24/29; 65.5–92.4) | 100.0  (17/17; 81.6–100.0) | 58.3  (7/12; 32.0–80.7) |
| Cumulative Cyto | NA | 81.8  (18/22; 61.5–92.7) | 100.0  (7/7; 64.6–100.0) | 86.2  (25/29; 69.4–94.5) | 100.0  (18/18; 82.4–100.0) | 63.6  (7/11; 35.4–84.8) |
| sEV-miRNA panel | 1.000  (1.000–1.000) | 100.0  (22/22; 85.1–100.0) | 100.0  (7/7; 64.6–100.0) | 100.0  (29/29; 88.3–100.0) | 100.0  (22/22; 85.1–100.0) | 100.0  (7/7; 64.6–100.0) |

**Note:** Cumulative MRI and Cumulative CSF cytology refer to all available MRI or CSF cytology assessments during clinical follow-up; patients were considered positive if any available assessment was positive. The sEV-miRNA panel was evaluated based on a single CSF sample per patient; for patients with repeat CSF sampling (C5, C10, C11, and C17), only the first available sample was used for all patient-level diagnostic performance calculations. Abbreviations: LM, leptomeningeal metastasis; CSF, cerebrospinal fluid; sEV, small extracellular vesicle; TP, true positive; TN, true negative; ACC, accuracy; SEN, sensitivity; SPE, specificity; AUC, area under the receiver-operating-characteristic curve; PPV, positive predictive value; NPV, negative predictive value; CI, confidence interval.

**Supplementary Table S4. Diagnostic yield of initial and cumulative conventional assessments and the single-sample sEV-miRNA panel relative to final adjudicated LM status in the validation cohort.**

| **Diagnostic method** | **AUC (95% CI)** | **Sensitivity, % (n/N; 95% CI)** | **Specificity, % (n/N; 95% CI)** | **Accuracy, %**  **(n/N; 95% CI)** | **PPV, %**  **(n/N; 95% CI)** | **NPV, %**  **(n/N; 95% CI)** |
| --- | --- | --- | --- | --- | --- | --- |
| Initial MRI | NA | 50.0  (10/20; 29.9–70.1) | 100.0  (16/16; 80.6–100.0) | 72.2  (26/36; 56.0–84.2) | 100.0  (10/10; 72.2–100.0) | 61.5  (16/26; 42.5–77.6) |
| Initial Cyto | NA | 77.3  (17/22; 56.6–89.9) | 100.0  (16/16; 80.6–100.0) | 86.8  (33/38; 72.7–94.2) | 100.0  (17/17; 81.6–100.0) | 76.2  (16/21; 54.9–89.4) |
| Cumulative MRI | NA | 70.0  (14/20; 48.1–85.5) | 93.8  (15/16; 71.7–98.9) | 80.6  (29/36; 65.0–90.2) | 93.3  (14/15; 70.2–98.8) | 71.4  (15/21; 50.0–86.2) |
| Cumulative Cyto | NA | 86.4  (19/22; 66.7–95.3) | 100.0  (16/16; 80.6–100.0) | 92.1  (35/38; 79.2–97.3) | 100.0  (19/19; 83.2–100.0) | 84.2  (16/19; 62.4–94.5) |
| sEV-miRNA panel | 0.935  (0.850–1.000) | 86.4  (19/22; 66.7–95.3) | 87.5  (14/16; 64.0–96.5) | 86.8  (33/38; 72.7–94.2) | 90.5  (19/21; 71.1–97.3) | 82.4  (14/17; 59.0–93.8) |

**Note:** Values are shown as percentages (n/N; 95% CI), unless otherwise indicated. Initial MRI and Initial CSF cytology refer to the first available MRI and CSF cytology results during clinical evaluation for suspected LM. Cumulative MRI and Cumulative CSF cytology refer to all available MRI or CSF cytology assessments during clinical follow-up; patients were considered positive if any available assessment was positive. For MRI-based analyses, two LM+ cases without available MRI results were excluded from the MRI-specific denominators. The sEV-miRNA panel was evaluated using a single CSF sample from each patient and was not used for LM adjudication. AUC was reported for the sEV-miRNA panel because it generated a continuous panel probability score. AUC was not reported for MRI or CSF cytology because these assessments were analyzed as binary clinical test results rather than continuous scores. The 95% confidence interval for AUC was calculated using DeLong’s method. The 95% confidence intervals for sensitivity, specificity, accuracy, PPV, and NPV were calculated using the Wilson score method. Abbreviations: LM, leptomeningeal metastasis; CSF, cerebrospinal fluid; sEV, small extracellular vesicle; PPV, positive predictive value; NPV, negative predictive value; NA, not applicable.
